# Author Correction: Phosphorylation of Jhd2 by the Ras-cAMP-PKA(Tpk2) pathway regulates histone modifications and autophagy

**DOI:** 10.1038/s41467-025-57545-8

**Published:** 2025-04-10

**Authors:** Qi Yu, Xuanyunjing Gong, Yue Tong, Min Wang, Kai Duan, Xinyu Zhang, Feng Ge, Xilan Yu, Shanshan Li

**Affiliations:** 1 State Key Laboratory of Biocatalysis and Enzyme Engineering, School of Life Sciences, Hubei University, Wuhan, Hubei 430062 China; 2 Key Laboratory of Algal Biology, Institute of Hydrobiology, Chinese Academy of Sciences, Wuhan, Hubei 430072 China

**Keywords:** Autophagy, Epigenetics, Epigenetics, Enzymes

Correction to: *Nature Communications* 10.1038/s41467-022-33423-5, published online 27 September 2022

In the version of the article initially published, copy/paste errors during data conversion for figure preparation led to errors in the bar graphs shown for Jhd2 ChIP-qPCR at *PYK1* in *tpk2Δ* mutant in Fig. 7g, *ras1Δ* and *ras2Δ* mutants in Supplementary Fig. 1g, *ATG39* (WT SD-C) in Supplementary Fig. 10b, and in the Source Data for Supplementary Fig. 1g. For comparison, the original and corrected figures and Source Data are available in the Supplementary information accompanying this amendment. The errors are in presentation only and do not affect the conclusions of the article. The figures and Source Data are now updated in the HTML and PDF versions of the article.

## Supplementary information

Original and revised Fig. 7, Supplementary Figs. 1 and 10, Source Data Supplementary Fig. 1g

