# Supplementary material for: Phosphorylation of Jhd2 by the Ras-cAMP-PKA(Tpk2) pathway regulates histone modifications and autophagy
Source: Nat Commun. 2022 Sep 27;13:5675. doi: 10.1038/s41467-022-33423-5 (PMC9515143; doi:10.1038/s41467-022-33423-5)
Supplement: Supplementary file 1 — Supplementary Information [file 41467_2022_33423_MOESM1_ESM.pdf]

Supplementary Information

Phosphorylation of Jhd2 by the Ras-cAMP-PKA(Tpk2) pathway regulates histone modifications and autophagy

Qi Yu[1,3], Xuanyunjing Gong[1,3], Yue Tong[1], Min Wang[2], Kai Duan[1], Xinyu Zhang[1], Feng Ge[2], Xilan Yu[1,*], Shanshan Li[1,*]

[1]State Key Laboratory of Biocatalysis and Enzyme Engineering, School of Life Sciences, Hubei University, Wuhan, Hubei 430062, China

[2]Key Laboratory of Algal Biology, Institute of Hydrobiology, Chinese Academy of Sciences, Wuhan, Hubei 430072, China

[3]These authors contribute equally to this work

*Corresponding authors: Shanshan Li, shl@hubu.edu.cn

Xilan Yu, yuxilan@hubu.edu.cn

Supplementary Data:

1. Supplementary (Fig. 1-11).

2. Supplemental Tables (Table S1-2).

Supplementary Fig. 1

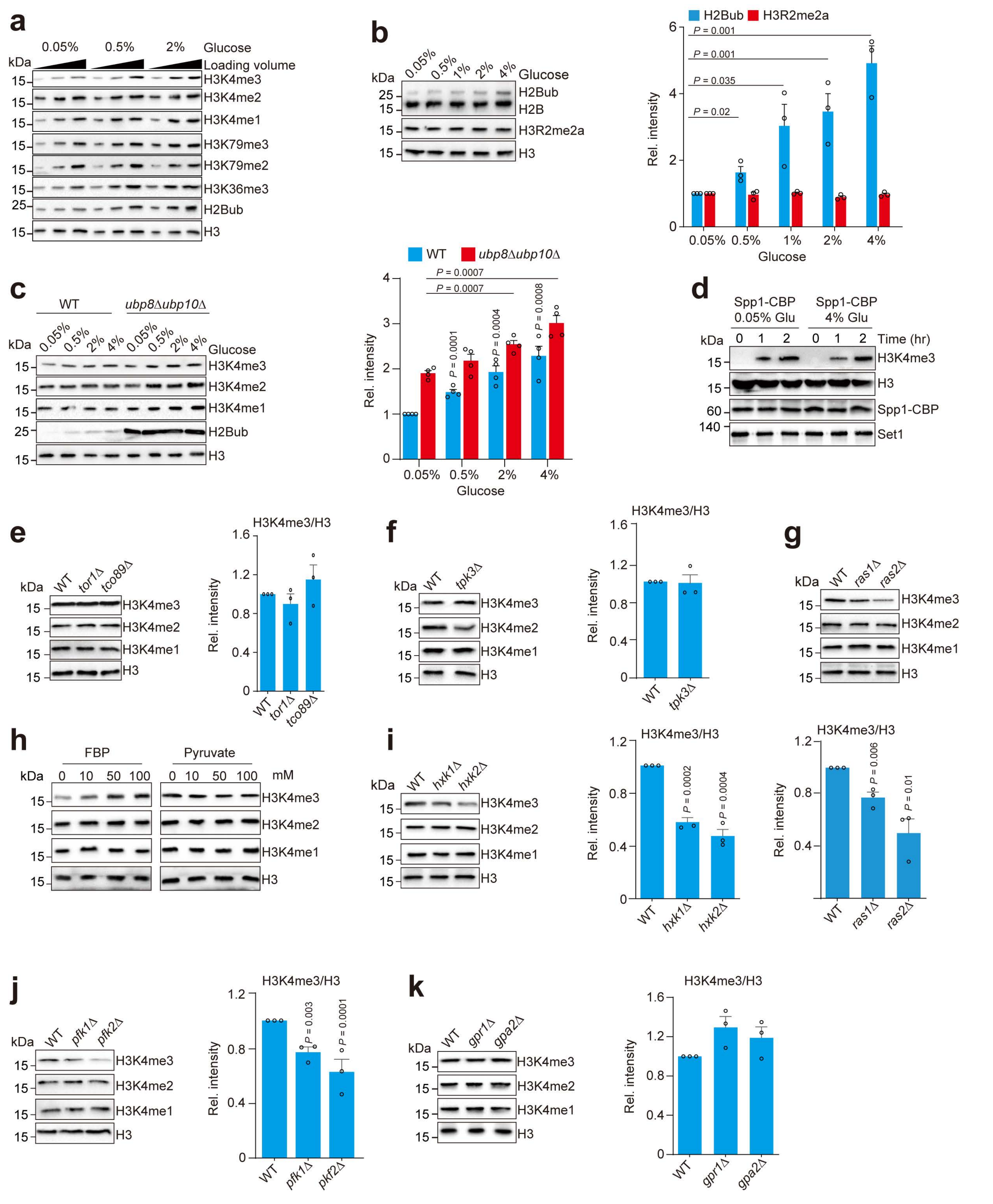

Supplementary Fig. 1 Glucose induces H3K4me3 via the Ras-cAMP-PKA(Tpk2) pathway.

a-b Effect of glucose on histone modifications. WT (a) and H2B-FLAG cells (b) were grown in YPD medium until OD_{600} of 0.7. Cells were then treated with YP medium supplemented with different concentrations of glucose for 0.5 hr. Cells were harvested and the extracted histones were analyzed by Western blots with indicated antibodies. To detect H2B monoubiquitination, H2B-FLAG strain was used and detected with anti-FLAG antibody. The upper bands are monoubiquitinated H2B-FLAG. **c** Effect of glucose on histone modifications in WT and *ubp8Δ ubp10Δ* mutant. WT and *ubp8Δ ubp10Δ* mutant were grown in YPD medium until OD_{600} of 0.7. Cells were then treated with YP medium supplemented with different concentrations of glucose for 0.5 hr. **d** Glucose has no effect on the activity of COMPASS. Spp1-TAP cells were grown in YPD medium until OD_{600} of 1.5. Cells were harvested and then treated with YP + 0.05% glucose and YP + 4% glucose for 0.5 hr. Spp1-containing COMPASS (Spp1-CBP) was then purified by tandem affinity purification (TAP). The *in vitro* histone methyltransferase assay was performed with purified COMPASS and *in vitro* assembled nucleosomes. **e** Effect of the mTOR pathway on H3K4 methylation. Cells were grown in YPD medium until OD_{600} of 0.7, harvested and the extracted histones were analyzed by Western blots with indicated antibodies. **f** Tpk3 has no effect on H3K4me3. **g** Effect of Ras1 and Ras2 on H3K4 methylation. **h** Fructose-1,6-biphosphate (FBP) but not pyruvate increases H3K4me3. **i-j** Effect of Hxk1, Hxk2, Pfk1 and Pfk2 on H3K4 methylation. **k** Western blot analysis of the effect of Gpr1 and Gpa2 on H3K4 methylation.

For Supplementary Fig. 1b, e-g, i-k, data represent means $\pm$ SEM; n=3 independent experiments. For Supplementary Fig. 1c, data represent means $\pm$ SEM; n=4 independent experiments. Two-sided t-tests were used for statistical analysis.

Supplementary Fig. 2

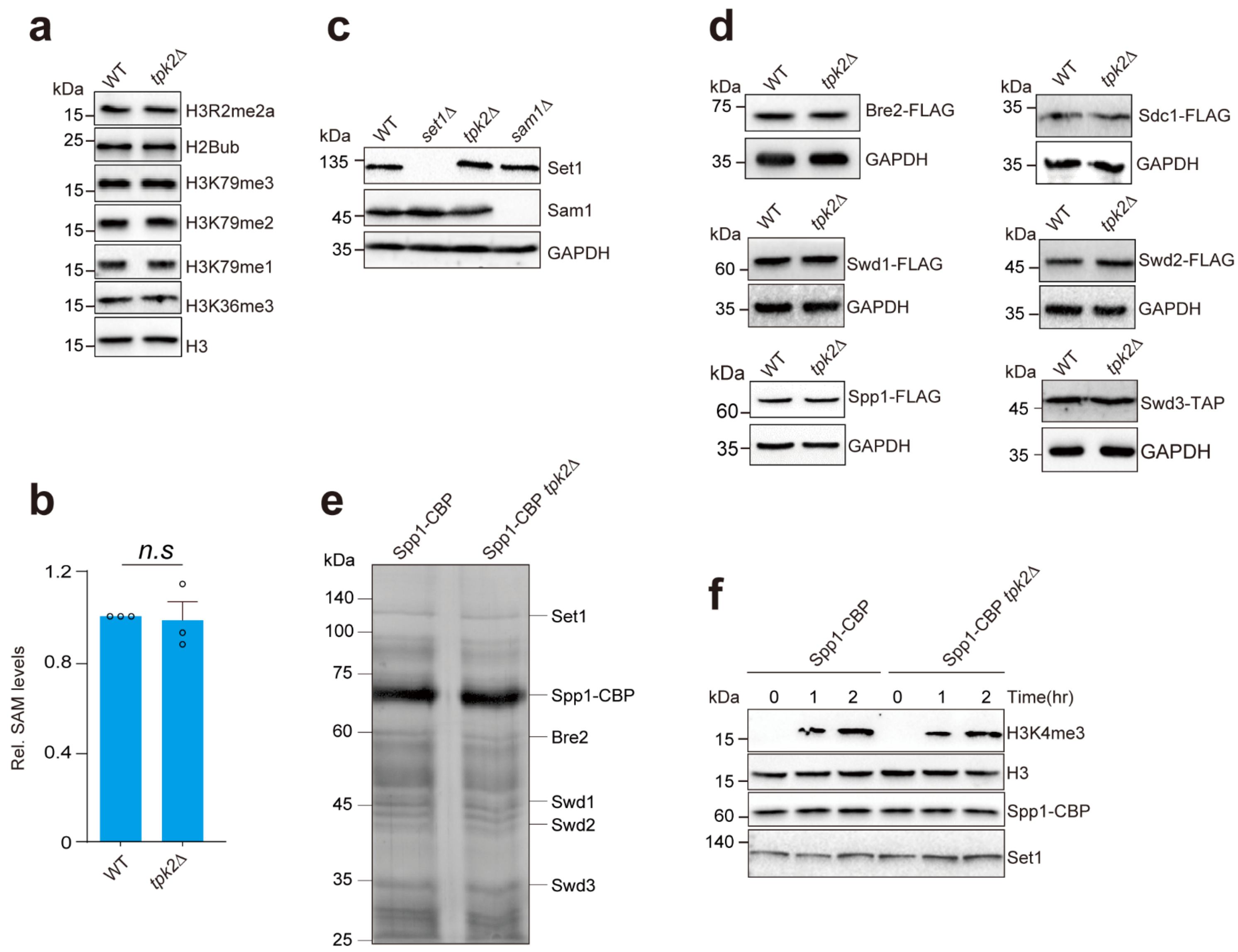

Supplementary Fig. 2 Effect of Tpk2 on the upstream regulators of H3K4me3.
a Effect of Tpk2 on histone modifications. WT and *tpk2Δ* mutant were grown in YPD medium until OD_{600} of 0.7, harvested and the extracted histones were analyzed by Western blots with indicated antibodies. **b** Effect of Tpk2 on intracellular SAM concentrations. WT and *tpk2Δ* mutant were grown in YPD medium until OD_{600} of 0.7. **c** Representative Western blot analysis of Set1 and Sam1 in WT and *tpk2Δ* mutant. **d** Western blot analysis of COMPASS subunits in WT and *tpk2Δ* mutant. Cells were grown in YPD medium until OD_{600} of 0.7. **e** Silver staining of COMPASS purified from WT and *tpk2Δ* mutant when grown in YPD medium until OD_{600} of 1. **f** The *in vitro* histone methyltransferase assay showing that Tpk2 had no effect on the activity of COMPASS.

For Supplementary Fig. 2b, data represent means ± SEM; n=3 independent experiments. *n.s,* no significance.

Supplementary Fig. 3

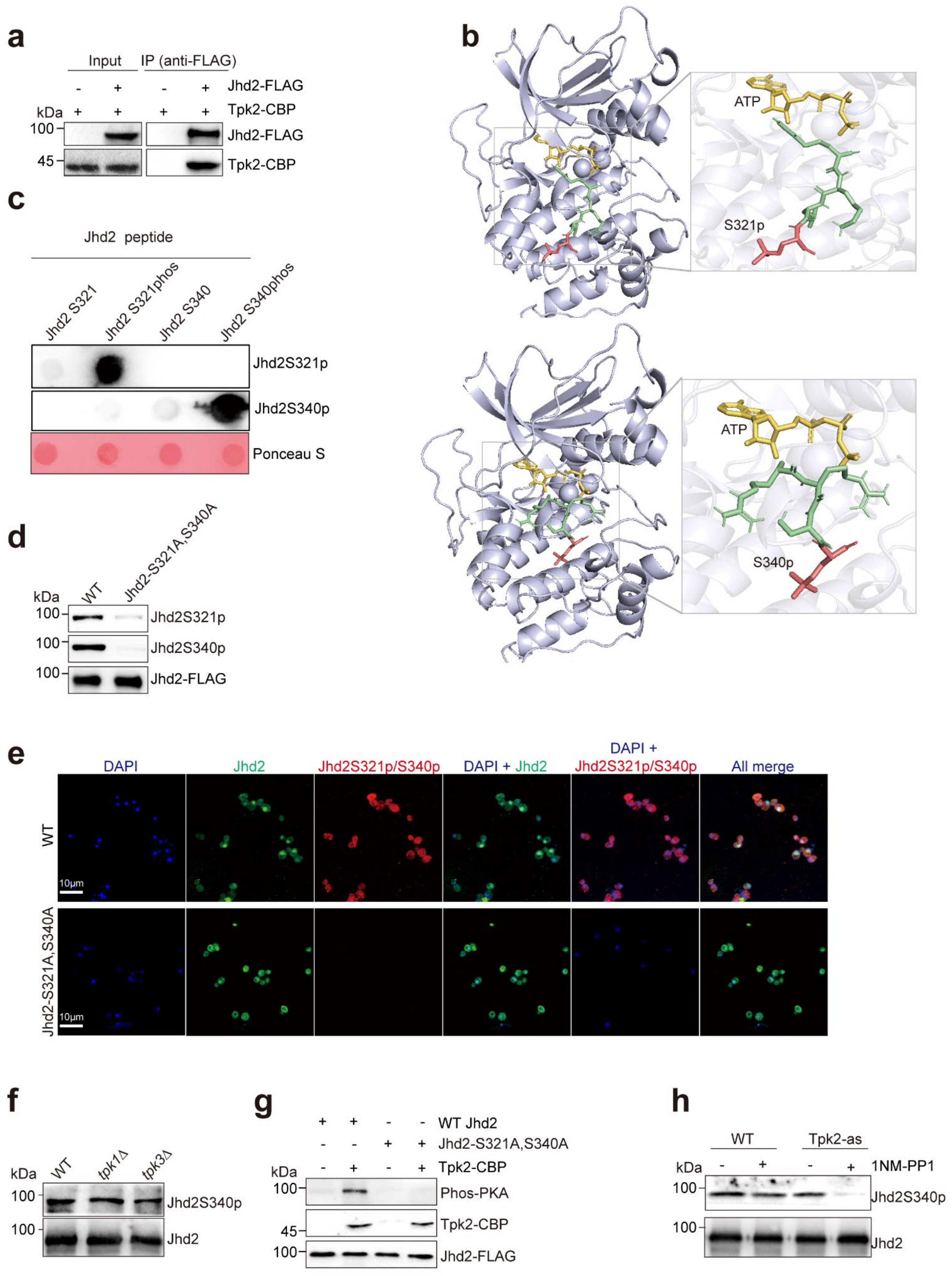

Supplementary Fig. 3 Tpk2 phosphorylates Jhd2 at serine (S321) and serine (S340) in response to glucose.

a *In vitro* Co-IP assay showing that Tpk2 directly interacted with Jhd2. Purified FLAG-tagged Jhd2 (Jhd2-FLAG) was incubated with TAP purified Tpk2 (Tpk2-CBP) at 4°C for 2 hr. Jhd2-FLAG was immunoprecipitated with anti-FLAG antibody and the co-IPed Tpk2 were detected by anti-CBP antibody. **b** Structural view of PKA subunit with phosphorylated Jhd2 peptides (318-RKLSp-321, 337-RRSSp-340) generated from molecular dynamic simulation. Jhd2 S321p and S340p are highlighted in red, respectively. **c** Dot blot analysis of the specificity of anti-Jhd2S321p and anti-Jhd2S340p antibodies with phosphorylated and unmodified peptides. 25 ng Jhd2 unmodified peptide (Jhd2 S321, Jhd2 S340) or phosphorylated Jhd2 peptides (Jhd2 S321phos, Jhd2 S340phos) were transferred to PVDF membrane and incubated with anti-Jhd2S321p and anti-Jhd2S340p antibodies. Ponceaus S staining was used a loading control. **d** Analysis of the specificity of anti-Jhd2S321p and anti-Jhd2S340p antibodies in WT and Jhd2-S321A, S340A mutant by Western blot analysis. **e** Analysis of the specificity of anti-Jhd2S321p and anti-Jhd2S340p antibodies in WT and Jhd2-S321A, S340A mutant by immunofluorescence. **f** Analysis of Jhd2 phosphorylation in WT, *tpk1Δ* and *tpk3Δ* mutants. **g** Tpk2 directly phosphorylates Jhd2 as determined by i*n vitro* kinase assay. **h** Analysis of Jhd2 phosphorylation in WT and Tpk2-as mutant when treated with or without 1NM-PP1. WT and Tpk2-as mutant were grown in YPD medium until OD_{600} of 0.7. Cells were then treated with 25 μM 1NM-PP1 for 0.5 hr.

Supplementary Fig. 4

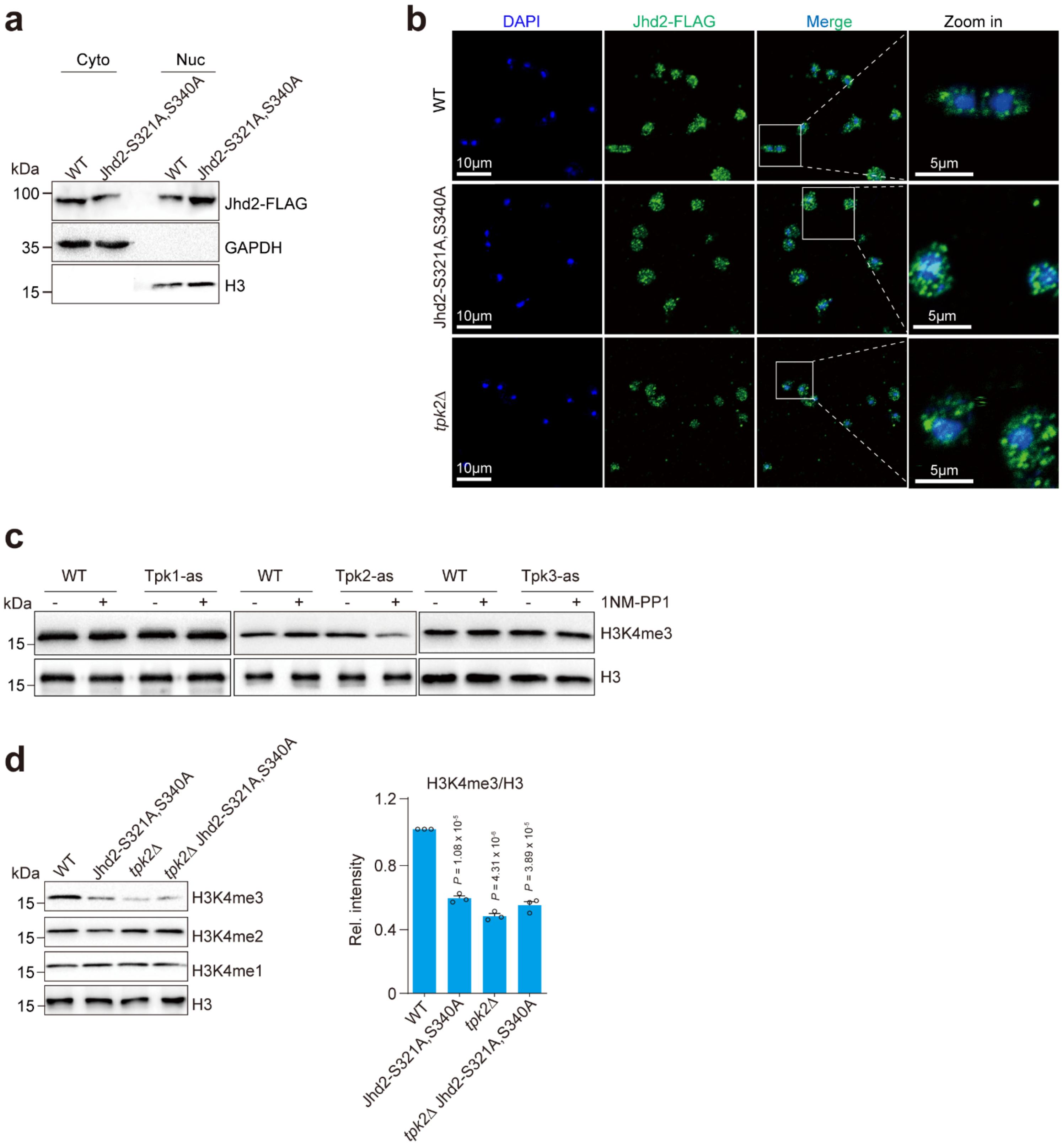

Supplementary Fig. 4 Tpk2-catalyzed Jhd2 phosphorylation reduces the nuclear localization and the enzymatic activity of Jhd2.

a Subcellular fractionation assay showing more Jhd2 occurred in the nucleus of Jhd2-S321A, S340A mutant. **b** Immunofluorescence showing more Jhd2 occurred in the nucleus of Jhd2-S321A, S340A and *tpk2Δ* mutants. **c** Analysis H3K4me3 in WT, Tpk1-as, Tpk2-as, and Tpk3-as mutants when treated with or without 1NM-PP1. WT, Tpk1-as, Tpk2-as, and Tpk3-as mutants were grown in YPD until medium until OD_{600} of 0.7. Cells were then treated with 25 μM 1NM-PP1 for 0.5 hr. **d** Western blot analysis of H3K4 methylation in WT, Jhd2-S321A, S340A , *tpk2*Δ, and *tpk2*Δ Jhd2-S321A, S340A mutants.

For Supplementary Fig. 4d, data represent means ± SEM; n=3 independent experiments. Two-sided t-tests were used for statistical analysis.

Supplementary Fig. 5

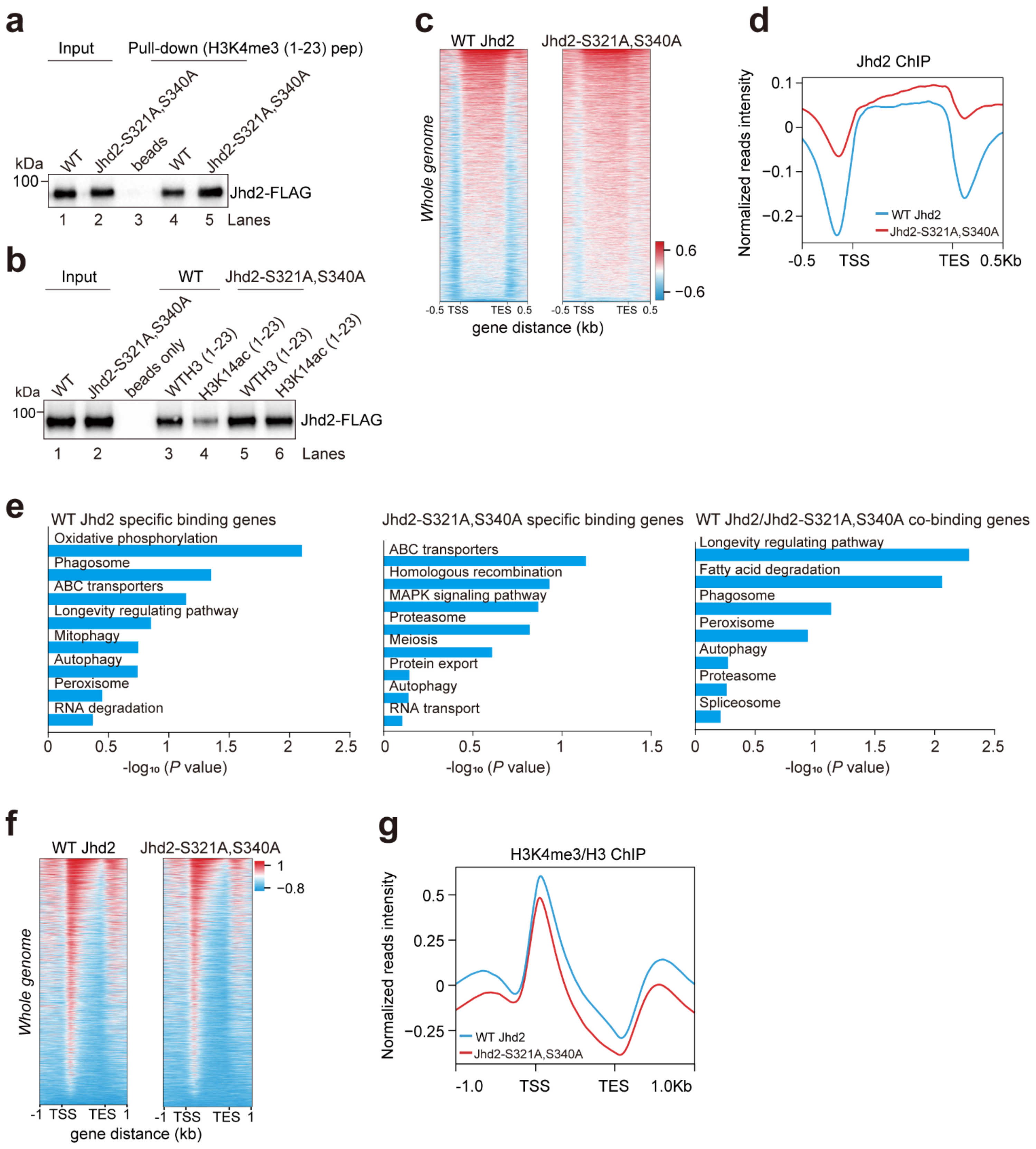

Supplementary Fig. 5 Tpk2-catalyzed Jhd2 phosphorylation reduces the binding of Jhd2 at chromatin.
a Peptide pull-down assay showing Jhd2-S321A, S340A had a higher binding affinity to H3K4me3 peptide (1-23) than WT Jhd2. 10 µg biotinylated H3K4me3 (1-23) peptides were incubated with 1 mg WT Jhd2 and Jhd2-S321A, S340A at 4°C overnight. The peptides and associated proteins were pull-down by incubation with Streptavidin beads at 4°C for 4 hr followed by Western blot analysis. **b** Peptide pull-down assay showing H3K14ac prevents the binding of WT Jhd2 but not Jhd2-S321A, S340A to H3 (1-23) peptide. **c** Distribution of Jhd2 binding across each gene through 0.5 kb upstream of the TSS to 0.5 kb downstream from the TES at all genes in WT and Jhd2-S321A, S340A mutant. **d** Averaged metagene profiles of Jhd2 binding in WT Jhd2 and Jhd2-S321A, S340A mutant. **e** KEGG analysis of WT Jhd2 specific binding genes, Jhd2-S321A, S340A specific binding genes, and WT Jhd2/Jhd2-S321A, S340A co-binding genes. One-side hypergeometric test was used for computing *P* values. **f, g** Averaged metagene profiles of H3K4me3/H3 enrichment in WT Jhd2 and Jhd2-S321A, S340A mutant.

Supplementary Fig. 6

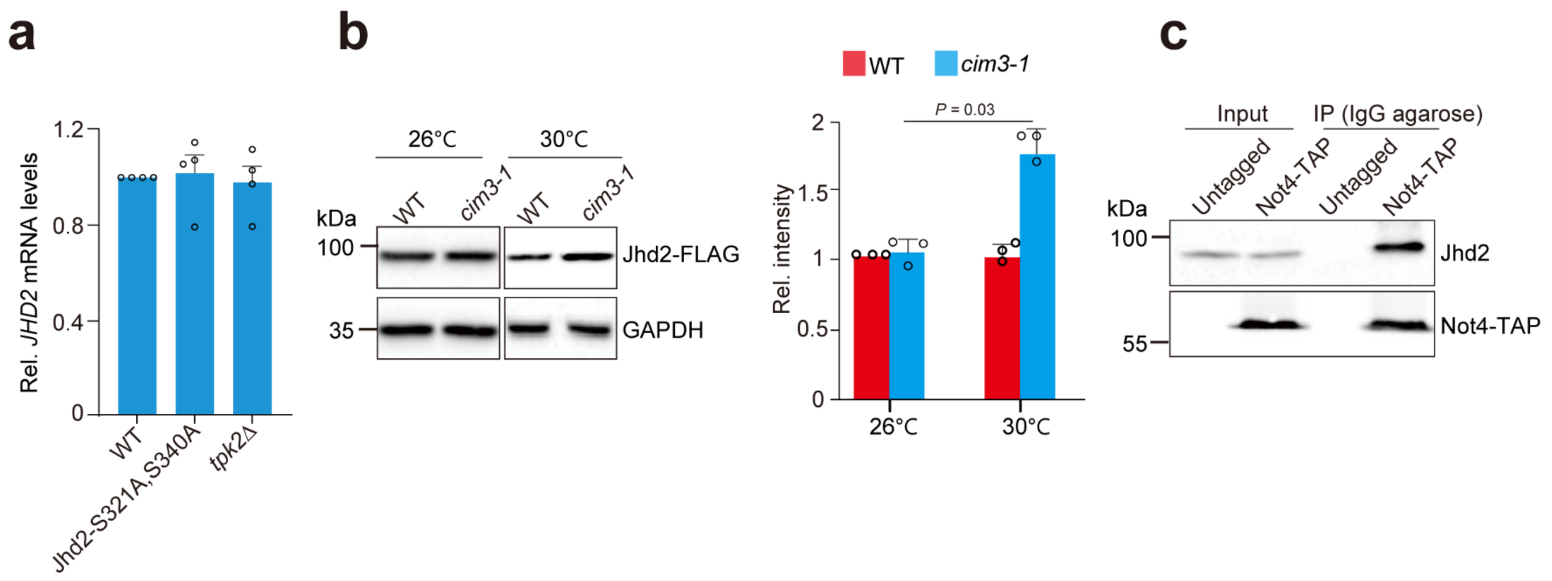

Supplementary Fig. 6 Tpk2-catalyzed Jhd2 phosphorylation promotes Jhd2 degradation by the proteasome pathway.
a RT-qPCR analysis of the relative *JHD2* mRNA levels in WT, Jhd2-S321A, S340A, and *tpk2Δ* mutants. **b** Jhd2 was degraded by the proteasome pathway. Inactivation of Cim3 in *Cim3*-1 mutant at 30℃ increased Jhd2 protein level. **c** Jhd2 interacted with Not4. TAP-tagged Not4 (Not4-TAP) was immunoprecipitated by IgG agarose beads and the co-IPed Jhd2 was detected by Western blots.

For Supplementary Fig. 6a, data represent means ± SEM; n=4 independent experiments. For Extended Data Fig. 6b, data represent means ± SEM; n=3 independent experiments. Two-sided t-tests were used for statistical analysis.

Supplementary Fig. 7

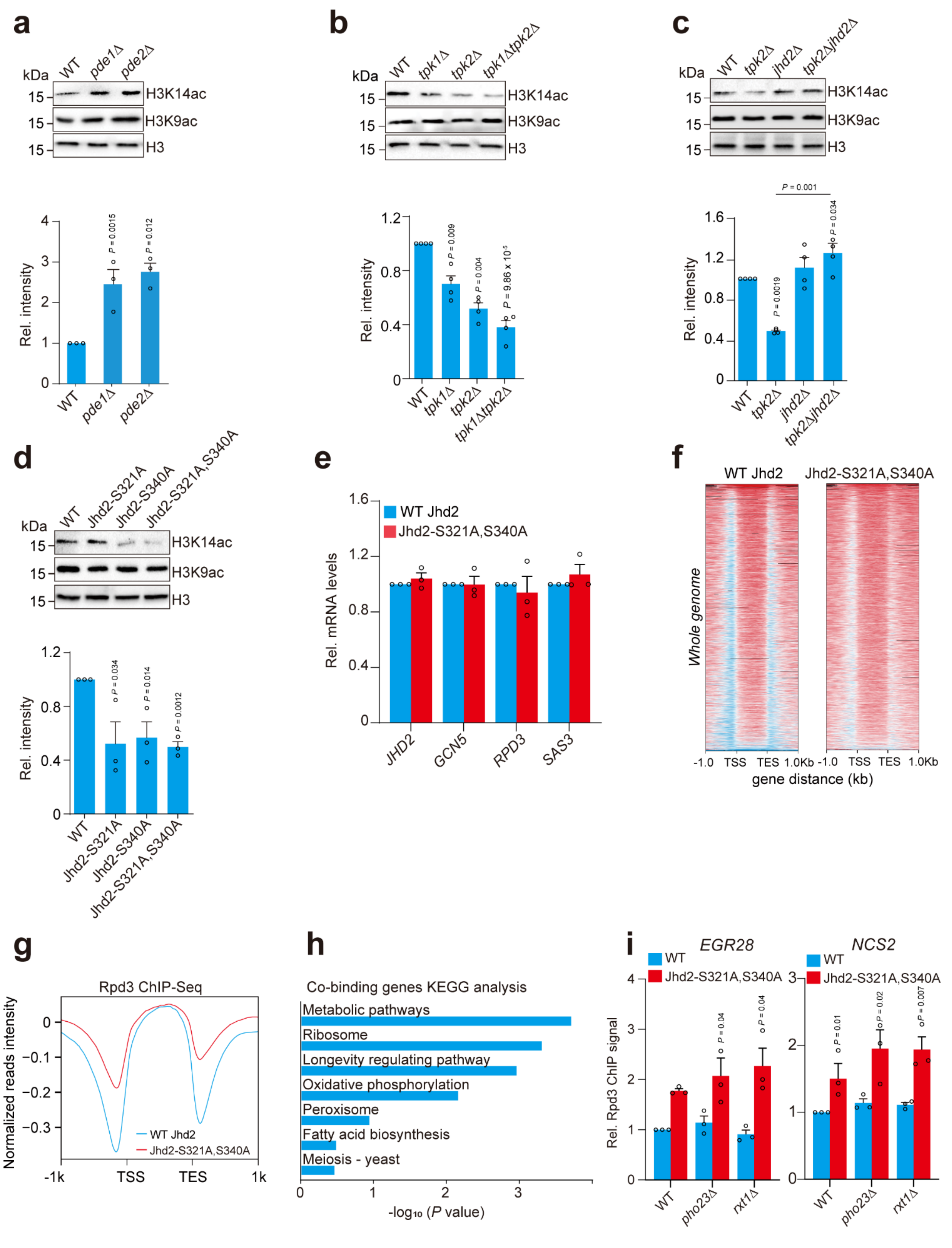

Supplementary Fig. 7 Tpk2-catalyzed Jhd2 phosphorylation maintains H3K14ac by preventing Rpd3 binding at chromatin.
a, b Analysis of H3K14ac and H3K9ac in WT, *pde1Δ*, *pde2Δ* , *tpk1Δ*, *tpk2Δ*, and *tpk1Δ tpk2Δ* mutants by Western blots. **c** Analysis of H3K14ac and H3K9ac in WT, *tpk2Δ*, *jhd2Δ*, and *tpk2Δ jhd2Δ* mutants by Western blots. **d** Analysis of H3K14ac and H3K9ac in WT, Jhd2-S321A, Jhd2-S340A and Jhd2-S321A, S340A mutants by Western blots. **e** Analysis of the transcription of *JHD2*, *GCN5*, *RPD3* and *SAS3* in WT and Jhd2-S321A, S340A mutant by RT-qPCR. **f** Distribution of Rpd3 binding across each gene through 1 kb upstream of the TSS to 1 kb downstream of the TES at all genes in WT Jhd2 and Jhd2-S321A, S340A mutant. **g** Averaged metagene profiles of Rpd3 binding in WT Jhd2 and Jhd2-S321A, S340A mutant. **h** KEGG analysis of Jhd2 and Rpd3 co-binding genes. One-side hypergeometric test was used for computing *P* values. **i** ChIP-qPCR analysis of Rpd3 occupancy at *EGR28* and *NCS2* in WT, *pho23Δ*, *rxt1Δ*, Jhd2-S321A, S340A, Jhd2-S321A, S340A *pho23Δ*, and Jhd2-S321A, S340A *rxt1Δ* mutants when cells were grown in YPD medium.

For Supplementary Fig. 7a, c-e, i data represent means ± SEM; n=3 independent experiments. For Extended Data Fig. 7b, data represent means ± SEM; n=4 independent experiments. Two-sided t-tests were used for statistical analysis.

Supplementary Fig. 8

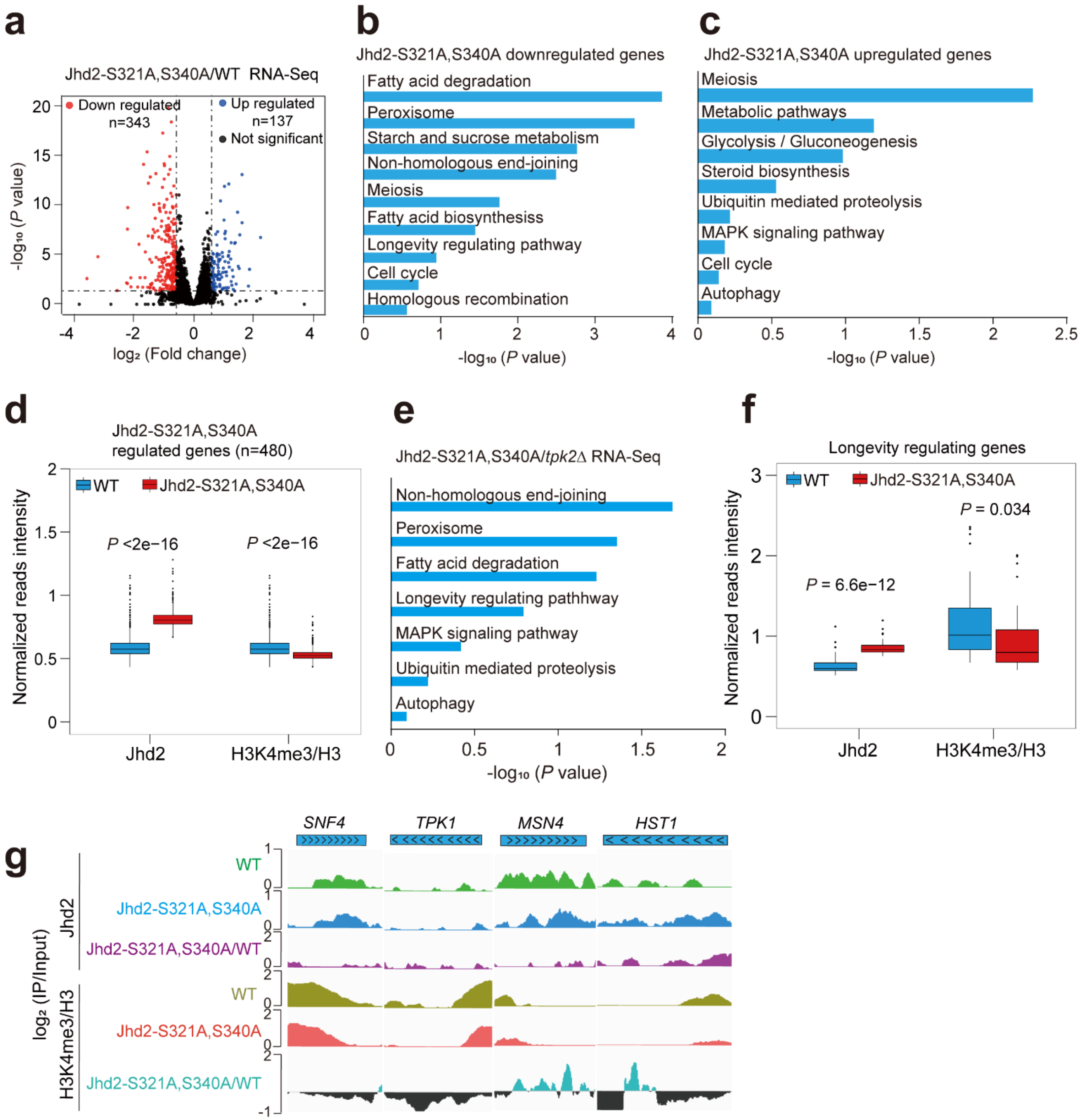

Supplementary Fig. 8 Tpk2-catalyzed Jhd2 phosphorylation regulates the transcription of genes involved in longevity regulating pathway.
a Volcano plots of RNA-seq data depicting differentially expressed genes in Jhd2-S321A, S340A mutant. Blue points designate upregulated genes and red points designate downregulated genes. **b, c** KEGG analysis of differentially expressed genes in Jhd2-S321A, S340A mutant. **d** Box plots showing that for 480 Jhd2-S321A, S340A-regulated genes, the occupancy of Jhd2 was significantly increased and H3K4me3 enrichment was significantly reduced in Jhd2-S321A, S340A mutant. **e** KEGG analysis of genes co-regulated in Jhd2-S321A, S340A and *tpk2Δ* mutants. **f** Box plots showing that for longevity regulating genes, the occupancy of Jhd2 was significantly increased and H3K4me3 enrichment was significantly reduced in Jhd2-S321A, S340A mutant. **g** ChIP-seq tracks showing the enrichment of Jhd2 and H3K4me3 at longevity regulating pathway genes.

For Supplementary Fig. 8d, f, centre lines denote medians; box limits denote 25th and 75th percentiles; whiskers denote maxima and minima. Two-sided Wilcoxon test in R (package ggpval) was used for statistical analysis. For Supplementary Fig. 8b, c, e, one-side hypergeometric test was used for computing *P* values.

Supplementary Fig. 9

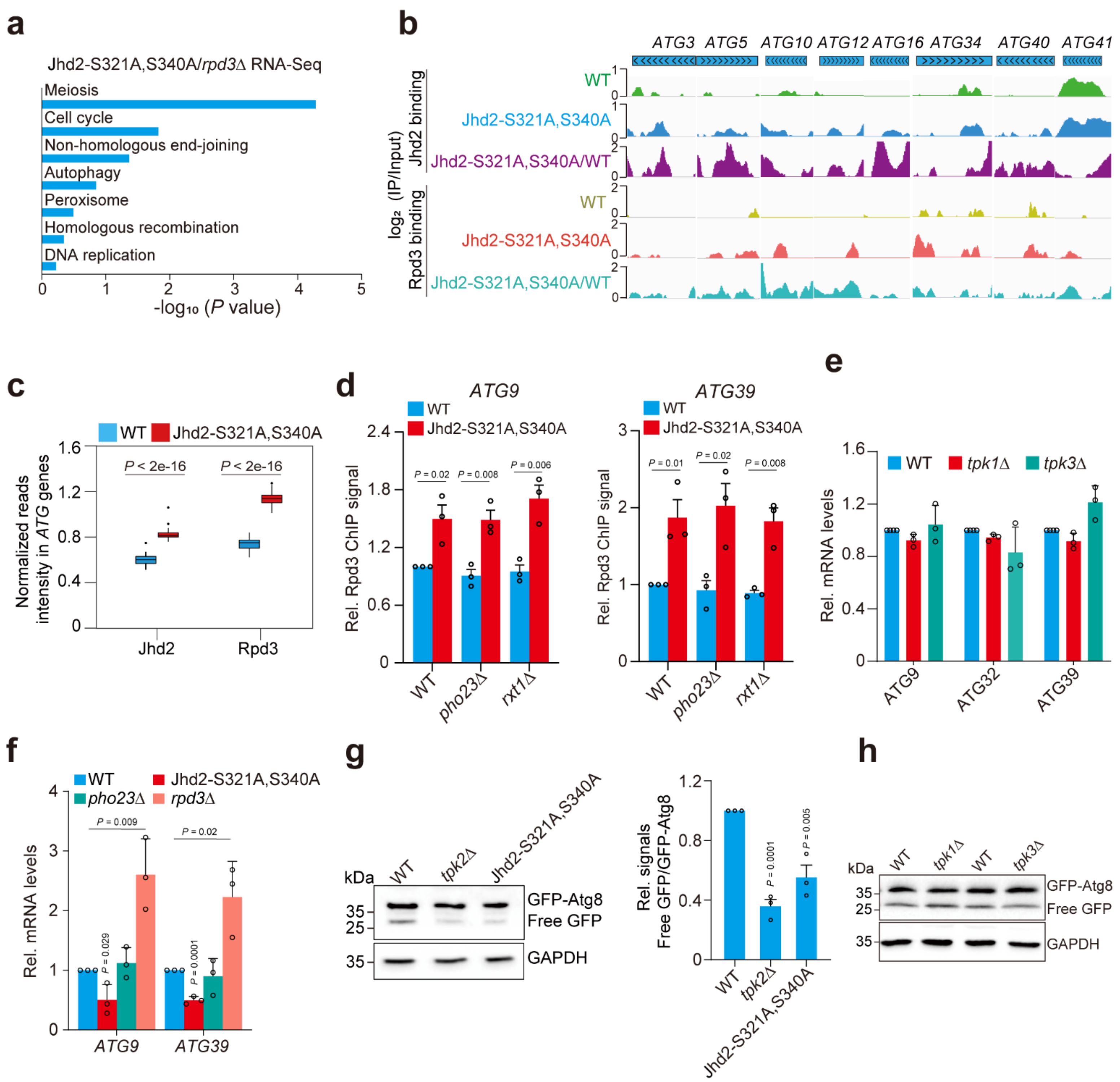

Supplementary Fig. 9 Tpk2-catalyzed Jhd2 phosphorylation promotes autophagy in part by inhibiting Rpd3. **a** KEGG analysis of genes co-regulated in Jhd2-S321A, S340A and *rpd3Δ* mutants. One-side hypergeometric test was used for computing *P* values. **b** ChIP-seq tracks showing the enrichment of Jhd2 and Rpd3 at autophagy genes in WT and Jhd2-S321A, S340A mutant. **c** Box plots showing the occupancy of Jhd2 and Rpd3 at all *ATG* genes was significantly increased in Jhd2-S321A, S340A mutant. **d** ChIP-qPCR analysis of Rpd3 occupancy at *ATG9* and *ATG39* in WT, *pho23Δ*, *rxt1Δ*, Jhd2-S321A, S340A, Jhd2-S321A, S340A *pho23Δ*, and Jhd2-S321A, S340A *rxt1Δ* mutants when cells were grown in YPD medium. **e** RT-qPCR analysis of the transcription of *ATG9*, *ATG32* and *ATG39* in WT, *tpk1Δ* and *tpk3Δ* mutants when cells were grown in YPD medium. **f** RT-qPCR analysis of the transcription of *ATG9* and *ATG39* in WT, Jhd2-S321A, S340A , *pho23Δ* and *rpd3Δ* mutants when cells were grown in YPD medium. **g** Representative immunoblot analysis of GFP-Atg8 and free GFP in WT, *tpk2Δ,* and Jhd2-S321A, S340A mutants with anti-GFP antibody. GAPDH was used as a loading control. **h** Representative immunoblot analysis of GFP-Atg8 and free GFP in WT, *tpk1Δ,* and *tpk3Δ* mutants with anti-GFP antibody.

For Supplementary Fig. 9d-g, data represent means ± SEM; n=3 independent experiments. Two-sided t-tests were used for statistical analysis. For Supplementary Fig. 9c, centre lines denote medians; box limits denote 25th and 75th percentiles; whiskers denote maxima and minima. Two-sided Wilcoxon test in R (package ggpval) was used for statistical analysis.

Supplementary Fig. 10

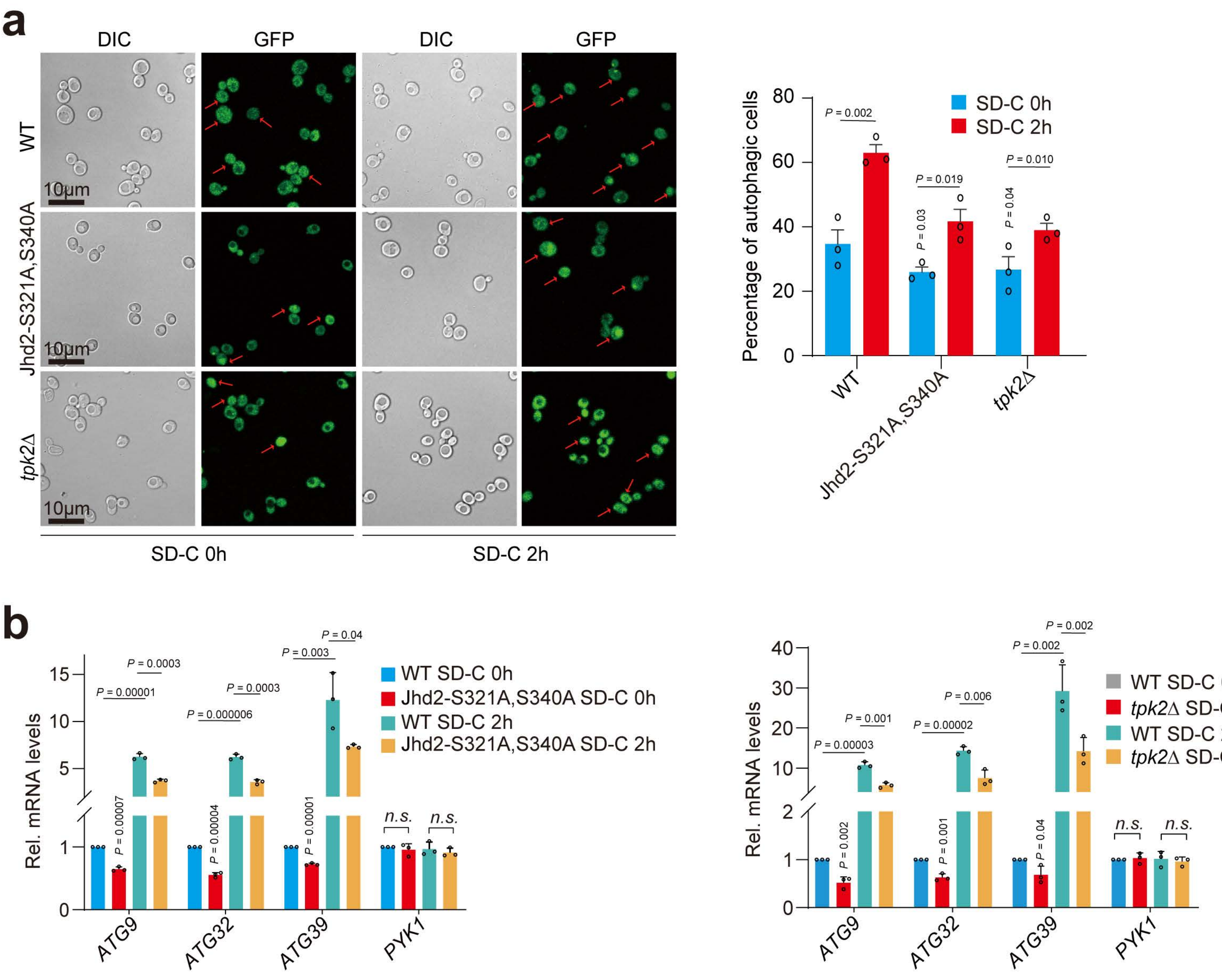

Supplementary Fig. 10 Tpk2-catalyzed Jhd2 phosphorylation promotes autophagy in part by inhibiting Rpd3 under glucose starvation conditions.

a Analysis of autophagy activity in WT, Jhd2-S321A, S340A, and *tpk2Δ* mutants when grown in glucose depletion medium (SD - C) for 0-2 hr by fluorescence assay. **b** Analysis of the transcription of *ATG9*, *ATG32*, and *ATG39* in WT, Jhd2-S321A, S340A, and *tpk2Δ* mutants when grown in glucose depletion medium (SD - C) for 0-2 hr by RT-qPCR.

For Supplementary Fig. 10a, b, data represent means ± SEM; n=3 independent experiments. Two-sided t-tests were used for statistical analysis.

Supplementary Fig. 11

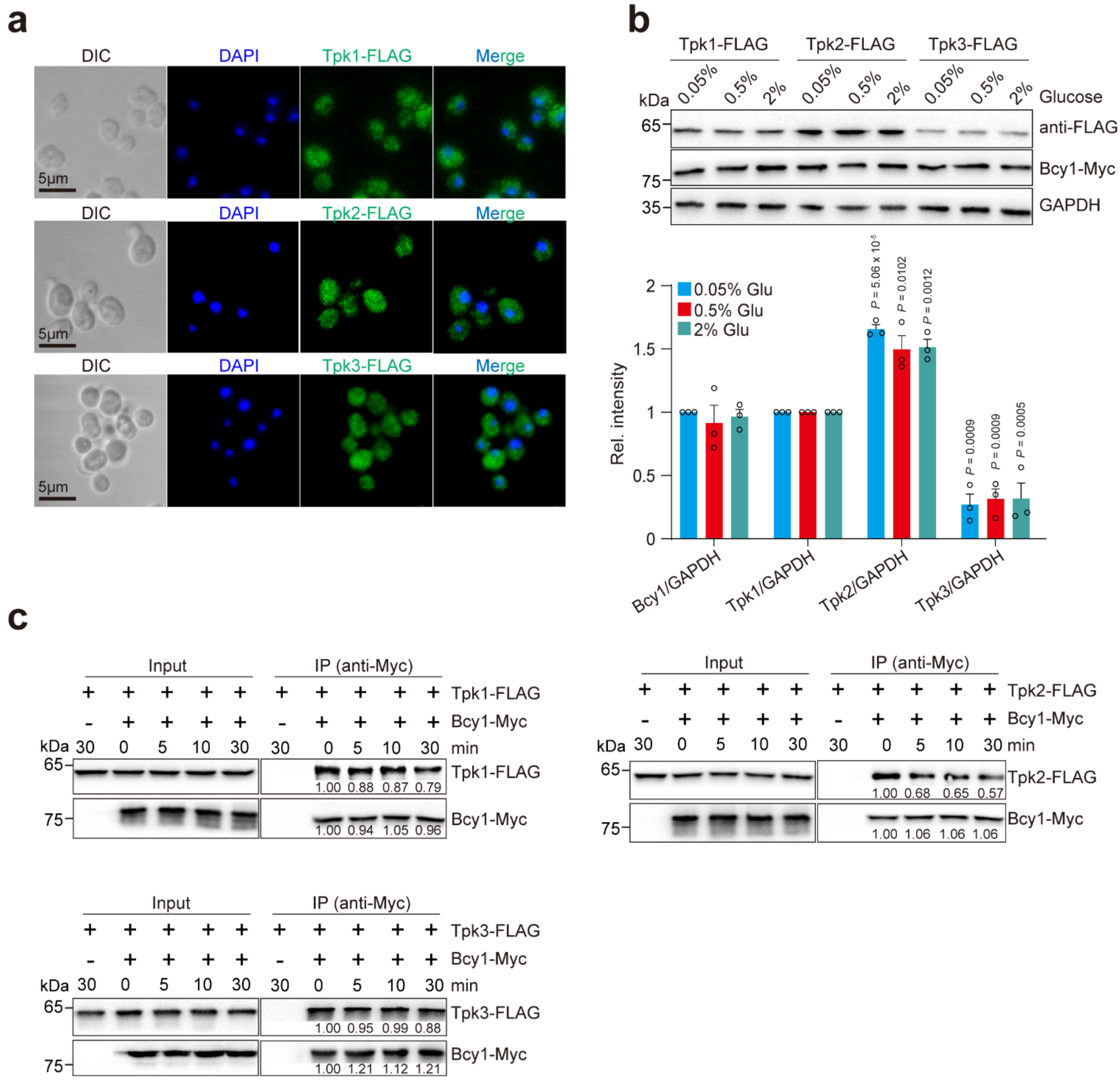

Supplementary Fig. 11 Analysis of PKA subunits subcellular localization, expression and Bcy1 dissociation kinetics.
a Subcellular localization of Tpk1, Tpk2 and Tpk3. Cells (Tpk1-FLAG, Tpk2-FLAG, Tpk3-FLAG) were grown in YP+2% glucose medium. The localization of Tpk1, Tpk2 and Tpk3 was detected with anti-FLAG antibody. DAPI was used to indicate the nucleus. **b** Analysis of the expression of Tpk1, Tpk2, Tpk3 and Bcy1 when cells were treated with 0.05%, 0.5% and 2% glucose by Western blots. **c** Analysis of the dissociation of Tpk1, Tpk2, Tpk3 with Bcy1 when cells were treated with 2% glucose for 0-30 min. Bcy1 was immunoprecipitated from cells (Tpk1-FLAG/Bcy1-Myc, Tpk2-FLAG/Bcy1-Myc, Tpk3-FLAG/Bcy1-Myc) with anti-Myc antibody.

For Supplementary Fig. 11b, data represent means ± SEM; n=3 independent experiments. Two-sided t-tests were used for statistical analysis.

Supplementary Table 1 List of strains used in this study

**Name**	**Parental Strain**	***Genotype***	**Source**
WT	BY4741	*MATa his3Δ1 leu2Δ0 met15Δ0 ura3Δ0*	Open Biosystems
snf1Δ	BY4741	*MATa his3Δ1 leu2Δ0 met15Δ0 ura3Δ0 snf1Δ::KAN*	Open Biosystems
sch9Δ	BY4741	*MATa his3Δ1 leu2Δ0 met15Δ0 ura3Δ0 sch9Δ::KAN*	Open Biosystems
tor1Δ	BY4741	*MATa his3Δ1 leu2Δ0 met15Δ0 ura3Δ0 tor1Δ::KAN*	Open Biosystems
ras2Δ	BY4741	*MATa his3Δ1 leu2Δ0 met15Δ0 ura3Δ0 ras2Δ::KAN*	Open Biosystems
tpk2Δ	BY4741	*MATa his3Δ1 leu2Δ0 met15Δ0 ura3Δ0 tpk2Δ::KAN*	Open Biosystems
tpk1Δ	BY4741	*MATa his3Δ1 leu2Δ0 met15Δ0 ura3Δ0 tpk1Δ::KAN*	Open Biosystems
tpk3Δ	BY4741	*MATa his3Δ1 leu2Δ0 met15Δ0 ura3Δ0 tpk3Δ::KAN*	Open Biosystems
tpk1Δ tpk2Δ	BY4741	*MATa his3Δ1 leu2Δ0 met15Δ0 ura3Δ0 tpk1Δ::KAN tpk2Δ::HIS3*	In this study
gpa2Δ	BY4741	*MATa his3Δ1 leu2Δ0 met15Δ0 ura3Δ0 gpa2Δ::KAN*	Open Biosystems
tco89Δ	BY4741	*MATa his3Δ1 leu2Δ0 met15Δ0 ura3Δ0 tco89Δ::KAN*	Open Biosystems
pde1Δ	BY4741	*MATa his3Δ1 leu2Δ0 met15Δ0 ura3Δ0 pde1Δ::KAN*	Open Biosystems
pde2Δ	BY4741	*MATa his3Δ1 leu2Δ0 met15Δ0 ura3Δ0 pde2Δ::KAN*	Open Biosystems
ras1Δ	BY4741	*MATa his3Δ1 leu2Δ0 met15Δ0 ura3Δ0 ras1Δ::KAN*	Open Biosystems
gpr1Δ	BY4741	*MATa his3Δ1 leu2Δ0 met15Δ0 ura3Δ0 gpr1Δ::KAN*	Open Biosystems
hxk1Δ	BY4741	*MATa his3Δ1 leu2Δ0 met15Δ0 ura3Δ0 hxk1Δ::KAN*	Open Biosystems
hxk2Δ	BY4741	*MATa his3Δ1 leu2Δ0 met15Δ0 ura3Δ0 hxk2Δ::KAN*	Open Biosystems
pfk1Δ	BY4741	*MATa his3Δ1 leu2Δ0 met15Δ0 ura3Δ0 pfk1Δ::KAN*	Open Biosystems
pfk2Δ	BY4741	*MATa his3Δ1 leu2Δ0 met15Δ0 ura3Δ0 pfk2Δ::KAN*	Open Biosystems
jhd2Δ	BY4741	*MATa his3Δ1 leu2Δ0 met15Δ0 ura3Δ0 jhd2Δ::KAN*	Open Biosystems

chd1Δ	BY4741	*MATa his3Δ1 leu2Δ0 met15Δ0 ura3Δ0 chd1Δ::KAN*	Open Biosystems
tpk2Δ jhd2Δ	BY4741	*MATa his3Δ1 leu2Δ0 met15Δ0 ura3Δ0 tpk2Δ::KAN jhd2Δ::URA3*	In this study
ubp8Δ ubp10Δ	BY4741	*MATa his3Δ1 leu2Δ0 met15Δ0 ura3Δ0 ubp8Δ::HIS3 ubp10Δ::LEU2*	In this study
Tpk2-TAP	BY4741	*MATa his3Δ1 leu2Δ0 met15Δ0 ura3Δ0 TPK2-TAP ::HIS3*	Open Biosystems
Jhd2-TAP	BY4741	*MATa his3Δ1 leu2Δ0 met15Δ0 ura3Δ0 JHD2-TAP ::HIS3*	Open Biosystems
Spp1-TAP	By4741	*MATa his3Δ1 leu2Δ0 met15Δ0 ura3Δ0 SPP1-TAP ::HIS3*	Open Biosystems
Spp1-TAP *tpk2Δ*	By4741	*MATa his3Δ1 leu2Δ0 met15Δ0 ura3Δ0 SPP1-TAP ::HIS3 tpk2Δ::LEU2*	In this study
Jhd2-S321A,S340A-TAP	BY4741	*MATa his3Δ1 leu2Δ0 met15Δ0 ura3Δ0 JHD2 S321A S340A-TAP ::KAN*	In this study
Rpd3-TAP	BY4741	*MATa his3Δ1 leu2Δ0 met15Δ0 ura3Δ0 RPD3-TAP ::HIS3*	Open Biosystems
WT Jhd2	BY4741	*MATa his3Δ1 leu2Δ0 met15Δ0 ura3Δ0 JHD2-3xFLAG::KAN*	In this study
Bre2-FLAG	BY4741	*MATa his3Δ1 leu2Δ0 met15Δ0 ura3Δ0 BRE2-3xFLAG::KAN*	In this study
Bre2-FLAG *tpk2Δ*	BY4741	*MATa his3Δ1 leu2Δ0 met15Δ0 ura3Δ0 BRE2-3xFLAG::KAN tpk2Δ::LEU2*	In this study
H2B-FLAG	BY4741	*MATa his3Δ1 leu2Δ0 met15Δ0 ura3Δ0 HTB2-3xFLAG::KAN*	In this study
H2B-FLAG *tpk2Δ*	BY4741	*MATa his3Δ1 leu2Δ0 met15Δ0 ura3Δ0 HTB2-3xFLAG::KAN tpk2Δ::LEU2*	In this study
Swd1-FLAG	BY4741	*MATa his3Δ1 leu2Δ0 met15Δ0 ura3Δ0 SWD1-3xFLAG::KAN*	In this study
Swd1-FLAG *tpk2Δ*	BY4741	*MATa his3Δ1 leu2Δ0 met15Δ0 ura3Δ0 SWD1-3xFLAG::KAN tpk2Δ::LEU2*	In this study
Swd2-FLAG	BY4741	*MATa his3Δ1 leu2Δ0 met15Δ0 ura3Δ0 SWD2-3xFLAG::KAN*	In this study
Swd2-FLAG *tpk2Δ*	BY4741	*MATa his3Δ1 leu2Δ0 met15Δ0 ura3Δ0 SWD2-3xFLAG::KAN tpk2Δ::LEU2*	In this study
Swd3-TAP	BY4741	*MATa his3Δ1 leu2Δ0 met15Δ0 ura3Δ0 SWD3-TAP ::HIS3*	In this study
Swd3-TAP *tpk2Δ*	BY4741	*MATa his3Δ1 leu2Δ0 met15Δ0 ura3Δ0 SWD3-TAP ::HIS3 tpk2Δ::LEU2*	In this study
Spp1-FLAG	BY4741	*MATa his3Δ1 leu2Δ0 met15Δ0 ura3Δ0 SPP1-3xFLAG::KAN*	In this study

Spp1-FLAG *tpk2Δ*	BY4741	*MATa his3Δ1 leu2Δ0 met15Δ0 ura3Δ0 SPP1-3xFLAG::KAN tpk2Δ::LEU2*	In this study
Sdc1-FLAG	BY4741	*MATa his3Δ1 leu2Δ0 met15Δ0 ura3Δ0 SDC1-3xFLAG::KAN*	In this study
Sdc1-FLAG *tpk2Δ*	BY4741	*MATa his3Δ1 leu2Δ0 met15Δ0 ura3Δ0 SDC1-3xFLAG::KAN tpk2Δ::LEU2*	In this study
Tpk1-FLAG	BY4741	*MATa his3Δ1 leu2Δ0 met15Δ0 ura3Δ0 TPK1-3xFLAG::KAN*	In this study
Tpk2-FLAG	BY4741	*MATa his3Δ1 leu2Δ0 met15Δ0 ura3Δ0 TPK2-3xFLAG::KAN*	In this study
Tpk3-FLAG	BY4741	*MATa his3Δ1 leu2Δ0 met15Δ0 ura3Δ0 TPK3-3xFLAG::KAN*	In this study
Tpk1-FLAG Bcy1-Myc	BY4741	*MATa his3Δ1 leu2Δ0 met15Δ0 ura3Δ0 TPK1-3xFLAG::KAN BCY1-13Myc:: HIS3*	In this study
Tpk2-FLAG Bcy1-Myc	BY4741	*MATa his3Δ1 leu2Δ0 met15Δ0 ura3Δ0 TPK2-3xFLAG::KAN BCY1-13Myc:: HIS3*	In this study
Tpk3-FLAG Bcy1-Myc	BY4741	*MATa his3Δ1 leu2Δ0 met15Δ0 ura3Δ0 TPK3-3xFLAG::KAN BCY1-13Myc:: HIS3*	In this study
Jhd2-S321A,S340A	BY4741	*MATa his3Δ1 leu2Δ0 met15Δ0 ura3Δ0 JHD2 S321A S340A-3xFLAG::HIS3*	In this study
WT Tpk1	BY4741	*MATa his3Δ1 leu2Δ0 met15Δ0 ura3Δ0 tpk1Δ::KAN [p413TEFpr-TPK1-HIS3]*	In this study
Tpk1-as	BY4741	*MATa his3Δ1 leu2Δ0 met15Δ0 ura3Δ0 tpk1Δ::KAN [p413TEFpr-TPK1 M164G-HIS3]*	In this study
WT Tpk2	BY4741	*MATa his3Δ1 leu2Δ0 met15Δ0 ura3Δ0 tpk2Δ::KAN [p413TEFpr-TPK2-HIS3]*	In this study
Tpk2-as	BY4741	*MATa his3Δ1 leu2Δ0 met15Δ0 ura3Δ0 tpk2Δ::KAN [p413TEFpr-TPK1 M147G-HIS3]*	In this study
WT Tpk3	BY4741	*MATa his3Δ1 leu2Δ0 met15Δ0 ura3Δ0 tpk3Δ::KAN [p413TEFpr-TPK3-HIS3]*	In this study
Tpk3-as	BY4741	*MATa his3Δ1 leu2Δ0 met15Δ0 ura3Δ0 tpk3Δ::KAN [p413TEFpr-TPK3 M165G-HIS3]*	In this study
tpk2Δ Jhd2-FLAG	BY4741	*MATa his3Δ1 leu2Δ0 met15Δ0 ura3Δ0 JHD2-3xFLAG::KAN tpk2Δ::URA3*	In this study
tpk2Δ Jhd2-S321A,S340A	BY4741	*MATa his3Δ1 leu2Δ0 met15Δ0 ura3Δ0 JHD2 S321A S340A-3xFLAG::HIS3 tpk2Δ::URA3*	In this study
set1Δ	BY4741	*MATa his3Δ1 leu2Δ0 met15Δ0 ura3Δ0 set1Δ::KAN*	Open Biosystems
sam1Δ	BY4741	*MATa his3Δ1 leu2Δ0 met15Δ0 ura3Δ0 sam1Δ::KAN*	Open Biosystems

WT+ *TEFpr-JHD2*	BY4741	*MATa his3Δ1 leu2Δ0 met15Δ0 ura3Δ0, jhd2Δ::URA3 [p413TEFpr-JHD2-HIS3]*	In this study
tpk2Δ+ TEFpr-JHD2	BY4741	*MATa his3Δ1 leu2Δ0 met15Δ0 ura3Δ0, jhd2Δ::URA3 tpk2Δ:: KAN [p413TEFpr-JHD2-HIS3]*	In this study
tpk2Δ+ TEFpr-JHD2 S321D	BY4741	*MATa his3Δ1 leu2Δ0 met15Δ0 ura3Δ0, jhd2Δ::URA3 tpk2Δ:: KAN [p413TEFpr-JHD2 S321D-HIS3]*	In this study
tpk2Δ+ TEFpr-JHD2 S340D	BY4741	*MATa his3Δ1 leu2Δ0 met15Δ0 ura3Δ0, jhd2Δ::URA3 tpk2Δ:: KAN [p413TEFpr-JHD2 S340D-HIS3]*	In this study
tpk2Δ+ TEFpr-JHD2-S321D,S340D	BY4741	*MATa his3Δ1 leu2Δ0 met15Δ0 ura3Δ0, jhd2Δ::URA3 tpk2Δ:: KAN [p413TEFpr-JHD2 S321D S340D-HIS3]*	In this study
Jhd2 S321A	BY4741	*MATa his3Δ1 leu2Δ0 met15Δ0 ura3Δ0, jhd2Δ::URA3 [p413TEFpr-JHD2 S321A-HIS3]*	In this study
Jhd2 S321D	BY4741	*MATa his3Δ1 leu2Δ0 met15Δ0 ura3Δ0, jhd2Δ::URA3 [p413TEFpr-JHD2 S321D-HIS3]*	In this study
Jhd2 S340A	BY4741	*MATa his3Δ1 leu2Δ0 met15Δ0 ura3Δ0, jhd2Δ::URA3 [p413TEFpr-JHD2 S340A-HIS3]*	In this study
Jhd2 S340D	BY4741	*MATa his3Δ1 leu2Δ0 met15Δ0 ura3Δ0, jhd2Δ::URA3 [p413TEFpr-JHD2 S340D-HIS3]*	In this study
Jhd2-S321D,S340D	BY4741	*MATa his3Δ1 leu2Δ0 met15Δ0 ura3Δ0, jhd2Δ::URA3 [p413TEFpr-JHD2 S321D S340D-HIS3]*	In this study
cim3-1	BY4741		Gift from Dr. Hai-Ning Du
Jhd2-S321A,S340A *cim3-1*	BY4741	*MATa his3Δ1 leu2Δ0 met15Δ0 ura3Δ0, JHD2 S321A S340A-3xFLAG::HIS3 CIM3-1*	In this study
not4Δ Jhd2-FLAG	BY4741	*MATa his3Δ1 leu2Δ0 met15Δ0 ura3Δ0 JHD2-3xFLAG::KAN not4Δ::LEU2*	In this study
not4Δ tpk2Δ Jhd2-FLAG	BY4741	*MATa his3Δ1 leu2Δ0 met15Δ0 ura3Δ0 JHD2-3xFLAG::KAN not4Δ::LEU2 tpk2Δ::URA3*	In this study
not4Δ Jhd2-S321A,S340A	BY4741	*MATa his3Δ1 leu2Δ0 met15Δ0 ura3Δ0 JHD2 S321A S340A-3xFLAG::HIS3 not4Δ::LEU2*	In this study
Not4-TAP	BY4741	*MATa his3Δ1 leu2Δ0 met15Δ0 ura3Δ0 NOT4-TAP::HIS3*	In this study

rpd3Δ Jhd2-S321A,S340A	BY4741	*MATa his3Δ1 leu2Δ0 met15Δ0 ura3Δ0 JHD2 S321A S340A-3xFLAG::HIS3 rpd3Δ::LEU2*	In this study
rpd3Δ Jhd2-FLAG	BY4741	*MATa his3Δ1 leu2Δ0 met15Δ0 ura3Δ0 JHD2-3xFLAG::KAN rpd3Δ::LEU2*	In this study
pho23Δ Jhd2-FLAG	BY4741	*MATa his3Δ1 leu2Δ0 met15Δ0 ura3Δ0 JHD2-3xFLAG::KAN pho23Δ::LEU2*	In this study
rxt1Δ Jhd2-FLAG	BY4741	*MATa his3Δ1 leu2Δ0 met15Δ0 ura3Δ0 JHD2-3xFLAG::KAN rxt1Δ::LEU2*	In this study
rpd3Δ tpk2Δ Jhd2-FLAG	BY4741	*MATa his3Δ1 leu2Δ0 met15Δ0 ura3Δ0 JHD2-3xFLAG::KAN rpd3Δ::LEU2 tpk2Δ::URA3*	In this study
WT Jhd2 ATG8p-GFP-Atg8	BY4741	*MATa his3Δ1 leu2Δ0 met15Δ0 ura3Δ0 JHD2-3xFLAG::KAN ATG8p-GFP-ATG8-URA3*	In this study
Jhd2-S321A,S340A ATG8p-GFP-Atg8	BY4741	*MATa his3Δ1 leu2Δ0 met15Δ0 ura3Δ0 JHD2 S321A S340A-3xFLAG::HIS3 ATG8p-GFP-ATG8-URA3*	In this study
tpk2Δ Jhd2-FLAG ATG8p-GFP-Atg8	BY4741	*MATa his3Δ1 leu2Δ0 met15Δ0 ura3Δ0 JHD2-3xFLAG::KAN tpk2Δ::LEU2 ATG8p-GFP-ATG8-URA3*	In this study
tpk1Δ ATG8p-GFP-Atg8	BY4741	*MATa his3Δ1 leu2Δ0 met15Δ0 ura3Δ0 ATG8p-GFP-ATG8-URA3 tpk1Δ::LEU2*	In this study
tpk3Δ ATG8p-GFP-Atg8	BY4741	*MATa his3Δ1 leu2Δ0 met15Δ0 ura3Δ0 ATG8p-GFP-ATG8-URA3 tpk3Δ::LEU2*	In this study
Tpk2-Flag	BY4741	*MATa his3Δ1 leu2Δ0 met15Δ0 ura3Δ0 TPK2-3xFLAG::KAN*	In this study
tpk1Δ Tpk2-Flag	BY4741	*MATa his3Δ1 leu2Δ0 met15Δ0 ura3Δ0 tpk1Δ::KAN TPK2-3xFLAG::URA3*	In this study
tpk3Δ Tpk2-Flag	BY4741	*MATa his3Δ1 leu2Δ0 met15Δ0 ura3Δ0 tpk3Δ::KAN TPK2-3xFLAG::URA3*	In this study

Supplementary Table 2 List of oligonucleotides used in this study

Gene name	Sequence
**qRT-PCR and ChIP-qPCR**	
PMA1	ACACTTTCGTTGGTAGAGCTG<br>AGAAACAAGCAGTCCAGACC
YEF3	ATGACCAAGGCTACCGAAAC<br>GGAGTAACTTCAGCAACGAAAG
ACTIN	TCGAACAAGAAATGCAAACCG<br>GGCAGATTCCAAACCCAAAAC
JHD2	GGAGCTTAGTGACCAAGAATCG<br>GGTAGTCTATCAATTCGTCCCC
ATG9	AGCAACTTCCCTTTACCAGAC<br>GTCAGACTCAGGAACACGTAAG
ATG32	CAGCATACGAACACCAAACAG<br>TGATTGTGTCGCTAGAGGAATC
ATG39	CTGGAGTTCCCACTATTTCAGAG<br>GTTTCGACCCCTCCATACTTG
PYK1	CCCAATCCCACCAAACCAC<br>TTCTACCAGCGGAGATGACCTT
EGR28	AGAACTTTCGGTACTTGGACC<br>CAGAGCCGAAGTGGAATAGG
NCS2	AGAAACTAATGTGAAGCCTAATTGC<br>TCGTTCTGAATATCCTGAAAGTATTTG
RPD3	TATGCCCCTAGTGTTCAGTTG<br>CACCCTTCGTATCTTTAGCCTC
SAS3	AGTGTTCGGTCTGATAAAGGC<br>CTGCTCCCATCTTATTGTCCC
GCN5	GCCCAAAGAATACATTGCCAG<br>GTTATGCCACCTACGACAGTC
REI1	ATCGTTCTTACCTGCCTTCG<br>TTGGTCTCTGTAGTGTGGTTG
TEA1	AGCAAAGTACAGCCCGTATC<br>TGAAATCCCATGTCGTAGCC
PAU8	GAATTGGGTGTCTACGTCTCTG<br>GGTAGGTTTCAGTTGGGTGG
DRS2	GGTGTCGGTATTAGTGGTATGG<br>TTGATAAGACCAGGAGCCATG
ATG5	ACCAGGTAAAGGATGTTCTCAC<br>TGCGATGGGAATGATAGTTGG
