# Supplementary material for: Phosphorylation of Jhd2 by the Ras-cAMP-PKA(Tpk2) pathway regulates histone modifications and autophagy
Source: Nat Commun. 2022 Sep 27;13:5675. doi: 10.1038/s41467-022-33423-5 (PMC9515143; doi:10.1038/s41467-022-33423-5)
Supplement: Supplementary file 3 — Reporting Summary [file 41467_2022_33423_MOESM3_ESM.pdf]

nature portfolio

Corresponding author(s): Shanshan Li

Last updated by author(s): 9/5/2022

Reporting Summary

Nature Portfolio wishes to improve the reproducibility of the work that we publish. This form provides structure for consistency and transparency in reporting. For further information on Nature Portfolio policies, see our Editorial Policies and the Editorial Policy Checklist.

Statistics

For all statistical analyses, confirm that the following items are present in the figure legend, table legend, main text, or Methods section.

n/a	Confirmed	
☐	☒	The exact sample size (*n*) for each experimental group/condition, given as a discrete number and unit of measurement
☐	☒	A statement on whether measurements were taken from distinct samples or whether the same sample was measured repeatedly
☐	☒	The statistical test(s) used AND whether they are one- or two-sided<br>*Only common tests should be described solely by name; describe more complex techniques in the Methods section.*
☒	☐	A description of all covariates tested
☒	☐	A description of any assumptions or corrections, such as tests of normality and adjustment for multiple comparisons
☐	☒	A full description of the statistical parameters including central tendency (e.g. means) or other basic estimates (e.g. regression coefficient) AND variation (e.g. standard deviation) or associated estimates of uncertainty (e.g. confidence intervals)
☐	☒	For null hypothesis testing, the test statistic (e.g. *F*, *t*, *r*) with confidence intervals, effect sizes, degrees of freedom and *P* value noted<br>*Give P values as exact values whenever suitable.*
☒	☐	For Bayesian analysis, information on the choice of priors and Markov chain Monte Carlo settings
☒	☐	For hierarchical and complex designs, identification of the appropriate level for tests and full reporting of outcomes
☒	☐	Estimates of effect sizes (e.g. Cohen's *d*, Pearson's *r*), indicating how they were calculated

Our web collection on statistics for biologists contains articles on many of the points above.

Software and code

Policy information about availability of computer code

Data collection	No software was used for data collection.
Data analysis	No custom software or algorithm was used in this study. All software used in this study for data analysis are either commercially available or open source.<br>Microsoft Excel (professional Plus2013) for basic statistical analysis<br>Proteome Discoverer 2.3 for AP-MS analysis (https://www.thermofisher.com/hk/en/home/industrial/mass-spectrometry/liquid-chromatography-mass-spectrometry-lc-ms/lc-ms-software/multi-omics-data-analysis/proteome-discoverer-software.html)<br>Image J (v.1.8.0)(for quantification of western blot images. https://imagej.en.softonic.com/)<br>ZEN 2.1(ZEN Imaging Software for microscope. https://www.zeiss.com.cn/microscopy/products/microscope-software/zen.html#inpagetabs-5)<br>prism8 for graphs (https://www.graphpad.com/scientific-software/prism/<br>SRA toolkit (v.2.9.2)(https://www.ncbi.nlm.nih.gov/sra/docs/toolkitsoft/)<br>Fastqc (v.0.11.9)(http://www.bioinformatics.babraham.ac.uk/projects/fastqc/)<br>Trim Galore (v.2.11)(https://github.com/FelixKrueger/TrimGalore)<br>Bowtie2 (v.2.1.0)(https://www.uio.no/english/services/it/research/hpc/abel/help/software/bowtie2.html)<br>Samtools (v.1.11)(https://github.com/samtools/samtools)<br>R (v.3.1.0)(https://www.r-project.org/)<br>EdgeR (v.3.24)(https://bioconductor.org/packages/release/bioc/html/edgeR.html)<br>MACS2 (v.2.1.1)(https://github.com/macs3-project/MACS)<br>deepTools2 (v.2.0) (https://deeptools.readthedocs.io/en/develop/content/installation.html)<br>IGV software (v.2.0)(http://software.broadinstitute.org/software/igv/download)

For manuscripts utilizing custom algorithms or software that are central to the research but not yet described in published literature, software must be made available to editors and reviewers. We strongly encourage code deposition in a community repository (e.g. GitHub). See the Nature Portfolio guidelines for submitting code & software for further information.

Data

Policy information about availability of data

All manuscripts must include a data availability statement. This statement should provide the following information, where applicable:

- Accession codes, unique identifiers, or web links for publicly available datasets
- A description of any restrictions on data availability
- For clinical datasets or third party data, please ensure that the statement adheres to our policy

All data supporting the findings of this study are included in the manuscript and its supplementary files are available.
The RNA-seq data for WT and Jhd2-S321A, S340A generated in this study have been deposited in the GEO database under accession number GSE175870 [https://www.ncbi.nlm.nih.gov/geo/query/acc.cgi?acc=GSE175870]. The ChIP-seq data for Jhd2, H3K4me3 and Rpd3 generated in this study have been deposited in the GEO database under accession number GSE175868 [https://www.ncbi.nlm.nih.gov/geo/query/acc.cgi?acc=GSE175868]. The RNA-seq data for rpd3 delta, jhd2 delta, and tpk2 delta are available in the GEO database under accession number GSE67149 [https://www.ncbi.nlm.nih.gov/geo/query/acc.cgi?acc=GSE67149], GSE73407 [https://www.ncbi.nlm.nih.gov/geo/query/acc.cgi?acc=GSE73407], and GSE28213 [https://www.ncbi.nlm.nih.gov/geo/query/acc.cgi?acc=GSE28213], respectively. The mass spectrometry proteomics data have been deposited to the ProteomeXchange Consortium via the PRIDE partner repository with the dataset identifier PXD030815 [http://proteomecentral.proteomexchange.org/cgi/GetDataset?ID=PXD030815]. The protein structure data for molecular docking used in this study is available on PDB database with identifier of 1atp (https://www.rcsb.org/structure/1ATP). The data that support the findings of this study are available from the corresponding authors upon reasonable request. Source data are provided with this paper.

Field-specific reporting

Please select the one below that is the best fit for your research. If you are not sure, read the appropriate sections before making your selection.

☒ Life sciences ☐ Behavioural & social sciences ☐ Ecological, evolutionary & environmental sciences

For a reference copy of the document with all sections, see nature.com/documents/nr-reporting-summary-flat.pdf

Life sciences study design

All studies must disclose on these points even when the disclosure is negative.

Sample size	Sample sizes were determined based on previous experience or similar published studies. To determine whether the outcome is statistically significant, at least three biological independent replicates were performed for each experiment. For other experiments, to determine the outcome is reproducible, at least 2-3 biological replicates were performed for each experiment. Related reference: Molecular Cell, 2015, 60:408-421; Nature Communications, 2021, 12: 594.
Data exclusions	No data were excluded from this study.
Replication	We confirmed that all attempts to replicate experiments were successful. All experiments were performed for at least 2-3 biological replicates, which were specified in the figure legends. To determine statistical significance, at least three biological replicates were used.
Randomization	Samples were allocated into groups by random.
Blinding	The investigators were blinded in design experiments and data analysis.

Reporting for specific materials, systems and methods

We require information from authors about some types of materials, experimental systems and methods used in many studies. Here, indicate whether each material, system or method listed is relevant to your study. If you are not sure if a list item applies to your research, read the appropriate section before selecting a response.

Materials & experimental systems

n/a	Involved in the study	
☐	☒	Antibodies
☒	☐	Eukaryotic cell lines
☒	☐	Palaeontology and archaeology
☒	☐	Animals and other organisms
☒	☐	Human research participants
☒	☐	Clinical data
☒	☐	Dual use research of concern

Methods

n/a	Involved in the study	
☐	☒	ChIP-seq
☒	☐	Flow cytometry
☒	☐	MRI-based neuroimaging

Antibodies

Antibodies used	Anti-H3 (9715S; Cell Signaling Technology)<br>Anti-H3 (1: 5000; ab1791. Abcam)<br>Anti-H4 (ab10158; Abcam)<br>Anti-H3K14ac (ab52946; Abcam)<br>Anti-H3K4me3 (A2357; Abclonal)<br>Anti-H3K4me2 (A2356; Abclonal)<br>Anti-H3K4me1 (A2355; Abclonal)<br>Anti-H3K36me3 (A2366; Abclonal)<br>Anti-H3K79me3 (A2369; Abclonal)<br>Alexa Fluor 488-conjugated Goat Anti-Mouse IgG (H+L)(AS037; Abclonal)<br>Alexa Fluor 594-conjugated Goat Anti-Rabbit IgG (H+L) (AS039; Abclonal)<br>Anti-Rpd3 (sc-514160; Santa Cruz Biotechnology)<br>Anti-Set1 (sc-101858; Santa Cruz Biotechnology)<br>Anti-GAPDH (10494-1-AP; proteintech)<br>Anti-GFP (66002-1-1g; proteintech)<br>Anti-Myc (60003-2-1g; proteintech)<br>goat polyclonal anti-mouse IgG (SA00001-1; proteintech)<br>goat polyclonal anti-rabbit IgG (SA00001-2; proteintech)<br>Anti-H3K9ac (9649S; Cell Signaling Technology)<br>Anti-H2B (12364S; Cell Signaling Technology)<br>Anti-H2Bub (5546S; Cell Signaling Technology)<br>Anti-phospho-PKA substrate (RRXS*/T*) (100G7E; Cell Signaling Technology)<br>Anti-FLAG M2 (F1804-1MG; Sigma-Aldrich)<br>Anti-FK2 (ST1200; Sigma-Aldrich)<br>Anti-CBP (Abs130593; Absin Bioscience Inc)<br>Anti-Jhd2 (1:500), Jhd2 S321p (1:500) and Jhd2 S340p (1:500) were custom-made in Abclonal<br>Anti-Sam1 was custom-made in Covance.
Validation	All antibodies have been validated for the application and species.<br>Anti-Histone H3 (9715S; Cell Signaling Technology) has been validated for Western blots and ChIP in yeast (PMID: 35880182).<br>Anti-H3 (1: 5000; ab1791. Abcam) has been validated for Western blots and ChIP in yeast (PMID: 33472063).<br>Anti-Histone H4 (ab10158; Abcam) has been validated for Western blots and ChIP in yeast (PMID: 27151365).<br>Anti-H3K14ac (ab52946; Abcam) has been validated for Western blots (PMID:32297950).<br>Anti-H3K4me3 (A2357; Abclonal) has been validated for Western blots and ChIP in yeast (PMID:31485071)<br>Anti-H3K4me2 (A2356; Abclonal) has ben validated for Western blots (PMID:31830521).<br>Anti-H3K4me1 (A2355; Abclonal) has ben validated for Western blots (PMID:31485071).<br>Anti-H3K36me3 (2366; Abclonal) has been validated for Western blots and ChIP in yeast (PMID:32619236).<br>Anti-H3K79me3 (A2369; Abclonal) has ben validated for Western blots (PMID:34824542).<br>Anti-Rpd3 (sc-514160; Santa Cruz Biotechnology) has been validated for Western blots in yeast (PMID:332663628).<br>Anti-Set1 (sc-101858; Santa Cruz Biotechnology) has been validated for Western blots in yeast (PMID:32358498).<br>Anti-GAPDH (10494-1-AP; proteintech) has been validated for Western blots (PMID:30759223).<br>Anti-GFP (66002-1-1g; proteintech) has been validated for Western blots of recombinant protein and GFP-tagged proteins (PMID:30699354).<br>Anti-Myc (60003-2-Ig; proteintech) has been validated for Western blots of recombinant protein and myc-tagged proteins (PMID:30869196).<br>goat polyclonal anti-mouse IgG (SA00001-1; proteintech) has been validated for Western blots(PMID:28901402).<br>goat polyclonal anti-rabbit IgG (SA00001-2; proteintech) has been validated for Western blots(PMID:30483785).<br>Anti-H3K9ac (9649S; Cell Signaling Technology) has been validated for Western blots in yeast (PMID:33154378).<br>Anti-H2B 12364S; Cell Signaling Technology) has been validated for Western blots (PMID:33257658).<br>Anti-H2Bub (5546S; Cell Signaling Technology) has been validated for Western blots (PMID:34473698).<br>Anti-phospho-PKA substrate (RRXS*/T*) (100G7E; Cell Signaling Technology) has been validated for Western blots (PMID:33175901).<br>Anti-Flag M2 (F1804-1MG; Sigma-Aldrich) has been validated for Western blots and ChIP in yeast (PMID:30759223).<br>Anti-FK2 (ST1200; Sigma-Aldrich) has been validated for Western blots (PMID: 26949039)<br>Anti-CBP (Abs130593; Absin Bioscience Inc) has been validated for Western blots (PMID: 34183849)<br>Anti-Sam1 has been validated for Western blots (PMID: 26527276).

ChIP-seq

Data deposition

☒ Confirm that both raw and final processed data have been deposited in a public database such as GEO.

☒ Confirm that you have deposited or provided access to graph files (e.g. BED files) for the called peaks.

Data access links<br>*May remain private before publication.*	The accession code for ChIP-seq of Jhd2,Rpd3 and H3K4me3 is GSE175868 (https://www.ncbi.nlm.nih.gov/geo/query/acc.cgi?acc=GSE175868). The accession number for WT Jhd2, Jhd2 S2A RNA-seq dataset is GSE175870 (https://www.ncbi.nlm.nih.gov/geo/query/acc.cgi?acc=GSE175870).

March 2021

Files in database submission	FASTQ files for ChIP-seq of anti-H3K4me3 and anti-H3,anti-FLAG,anti-Rpd3, Bigwig files, peak files, and data analysis files are also included.
Genome browser session (e.g. UCSC)	no longer applicable

Methodology

Replicates	One biological replicate for each ChIP-seq.
Sequencing depth	15M reads
Antibodies	Rabbit anti-H3 (ab1791; Abcam)<br>Rabbit anti-H3K4me3 (A2357; Abclonal)<br>Rabbit anti-FLAG M2 (F1804-1MG; Sigma-Aldrich)<br>anti-Rpd3 (sc-514160; Santa Cruz Biotechnology)
Peak calling parameters	Data analysis and peak calling parameters are provided in the methods. macs2 callpeak -t treatment.bam -c control.bam -g 1.2e7 -n -B -q 0.01 --nomodel
Data quality	All raw data used for analysis with Per base sequence quality greater than 30 (Q30 cutoff). All assigned peaks were identified based on an FDR < 0.001.
Software	All ChIP-seq data analysis software and methodology are described in the methods.<br>SRA toolkit (v.2.9.2)<br>Fastqc (v.0.11.9)<br>Trim Galore (v.0.3.1)<br>Bowtie2 (v.2.1.0)<br>Samtools (1.7-2)<br>MACS2 (v.2.1.1)<br>Bedtools (v.2.19.0)<br>R (v.3.1)<br>IGV<br>deeptools 2.0<br>hisat2 (v.2.1.0)<br>EdgeR (v.3.24)<br>ggplot2 (v.3.3.3)
