# Supplementary material for: Author Correction: Phosphorylation of Jhd2 by the Ras-cAMP-PKA(Tpk2) pathway regulates histone modifications and autophagy
Source: Nat Commun. 2025 Apr 10;16:3411. doi: 10.1038/s41467-025-57545-8 (PMC11986158; doi:10.1038/s41467-025-57545-8)

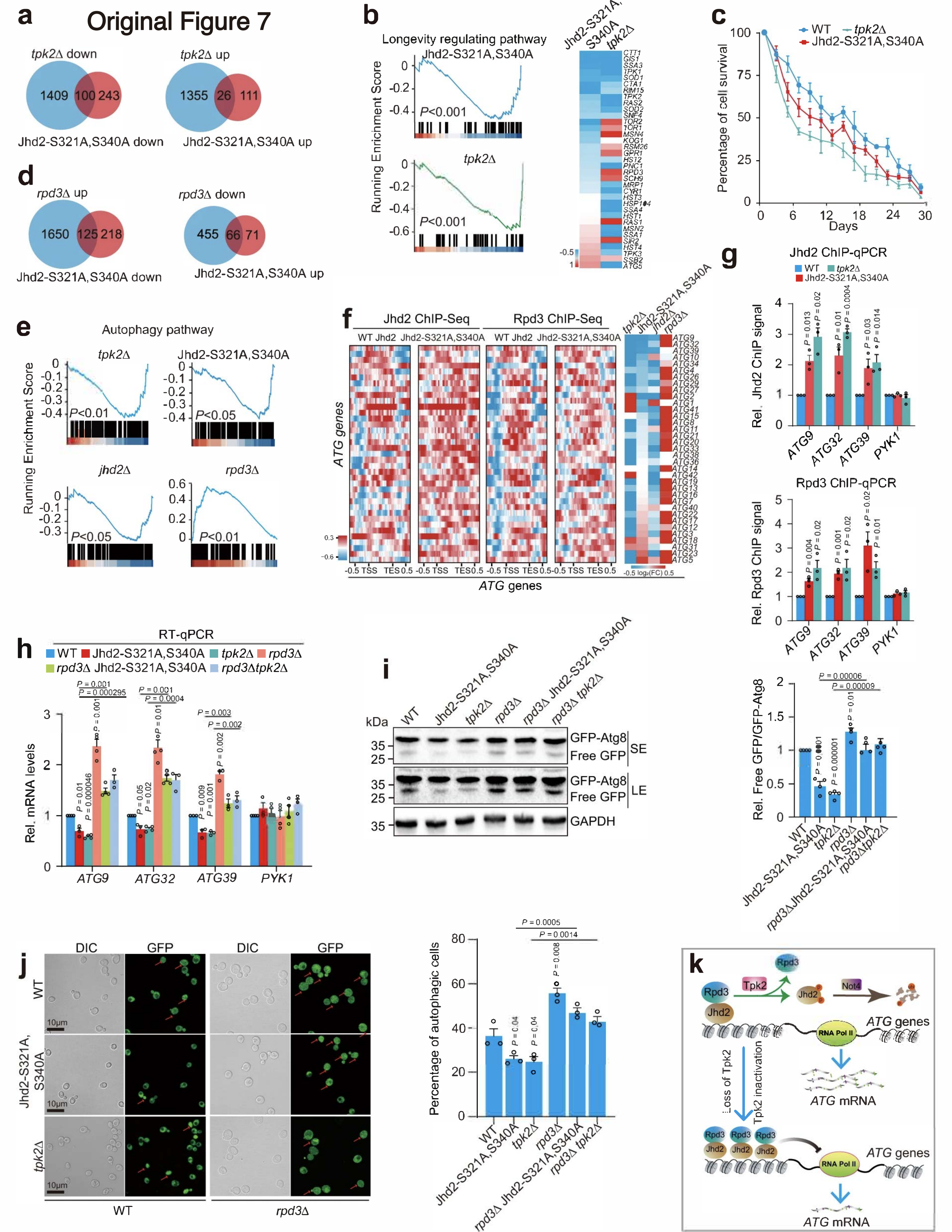

Original Figure 7
a
tpk2Δ down
1409 100 243
Jhd2-S321A,S340A down
tpk2Δ up
1355 26 111
Jhd2-S321A,S340A up
b
Longevity regulating pathway
Jhd2-S321A,S340A
P<0.001
tpk2Δ
P<0.001
Running Enrichment Score
Jhd2-S321A, S340A
tpk2Δ
CTT1
GIS1
SSA3
TPK1
SOD1
CTA1
RIM15
TPK2
RAS2
SOD2
SNF4
TOR2
TOR1
MSN4
KOG1
RSM26
GPR1
HS12
PNC1
RPD3
SCH9
MRP1
CYR1
HST3
HSP104
SSA4
HST1
RAS1
MSN2
SSA1
SIR2
HST4
TPK3
SSB2
ATG5
c
WT
tpk2Δ
Jhd2-S321A,S340A
Percentage of cell survival
Days
d
rpd3Δ up
1650 125 218
Jhd2-S321A,S340A down
rpd3Δ down
455 66 71
Jhd2-S321A,S340A up
e
Autophagy pathway
tpk2Δ
P<0.01
Jhd2-S321A,S340A
P<0.05
jhd2Δ
P<0.05
rpd3Δ
P<0.01
Running Enrichment Score
f
Jhd2 ChIP-Seq
Rpd3 ChIP-Seq
WT Jhd2
Jhd2-S321A,S340A
WT Jhd2
Jhd2-S321A,S340A
ATG genes
tpk2Δ
Jhd2-S321A,S340A
jhd2Δ
rpd3Δ
-0.5 TSS TES 0.5
-0.5 log2(FC) 0.5
ATG genes
g
Jhd2 ChIP-qPCR
WT
tpk2Δ
Jhd2-S321A,S340A
Rel. Jhd2 ChIP signal
P = 0.013
P = 0.02
P = 0.01
P = 0.0004
P = 0.03
P = 0.014
ATG9
ATG32
ATG39
PYK1
Rpd3 ChIP-qPCR
Rel. Rpd3 ChIP signal
P = 0.004
P = 0.02
P = 0.001
P = 0.02
P = 0.02
P = 0.01
ATG9
ATG32
ATG39
PYK1
h
RT-qPCR
WT
Jhd2-S321A,S340A
tpk2Δ
rpd3Δ
rpd3Δ Jhd2-S321A,S340A
rpd3Δtpk2Δ
Rel. mRNA levels
P = 0.001
P = 0.000295
P = 0.001
P = 0.0004
P = 0.003
P = 0.002
ATG9
ATG32
ATG39
PYK1
i
WT
Jhd2-S321A,S340A
tpk2Δ
rpd3Δ
rpd3Δ Jhd2-S321A,S340A
rpd3Δ tpk2Δ
kDa
GFP-Atg8
Free GFP
SE
LE
GAPDH
Rel. Free GFP/GFP-Atg8
P = 0.00006
P = 0.00009
P = 0.000001
j
DIC
GFP
WT
Jhd2-S321A, S340A
tpk2Δ
10μm
WT
rpd3Δ
Percentage of autophagic cells
P = 0.0005
P = 0.0014
P = 0.008
P = 0.04
k
Rpd3
Tpk2
Jhd2
Not4
ATG genes
RNA Pol II
ATG mRNA
Loss of Tpk2
Tpk2 inactivation

Revised Figure 7

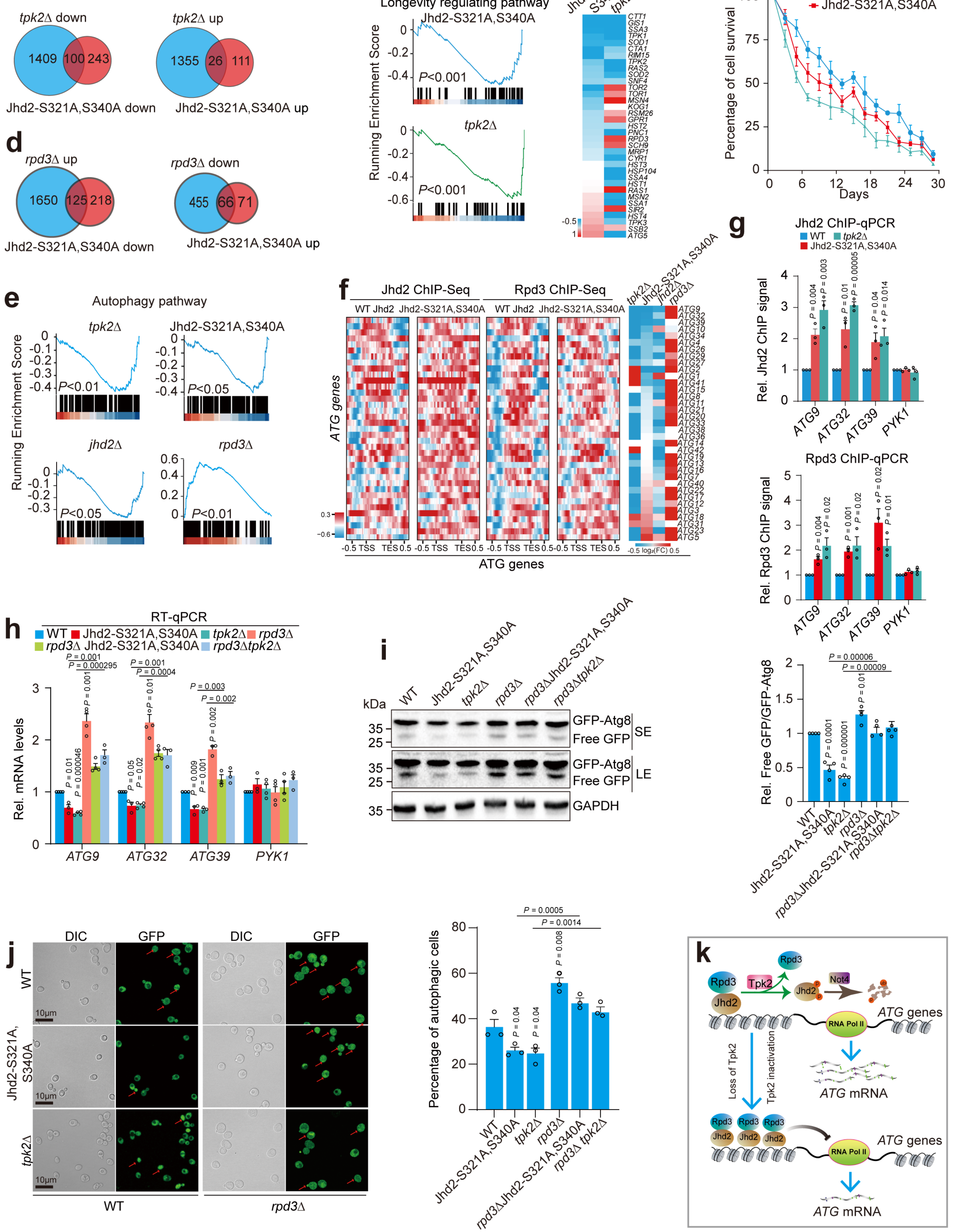

Original Supplementary Fig. 1

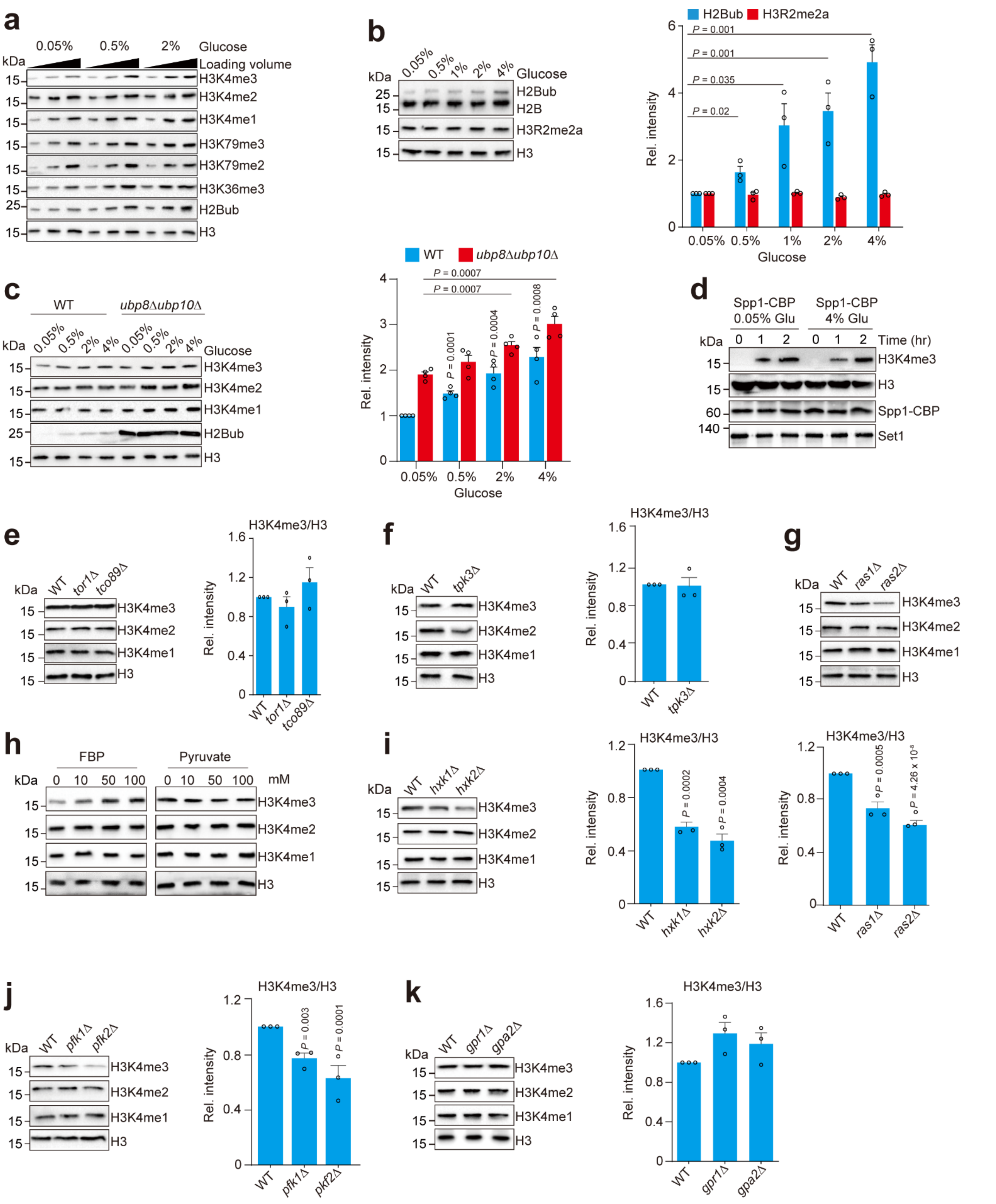

Revised Supplementary Fig. 1

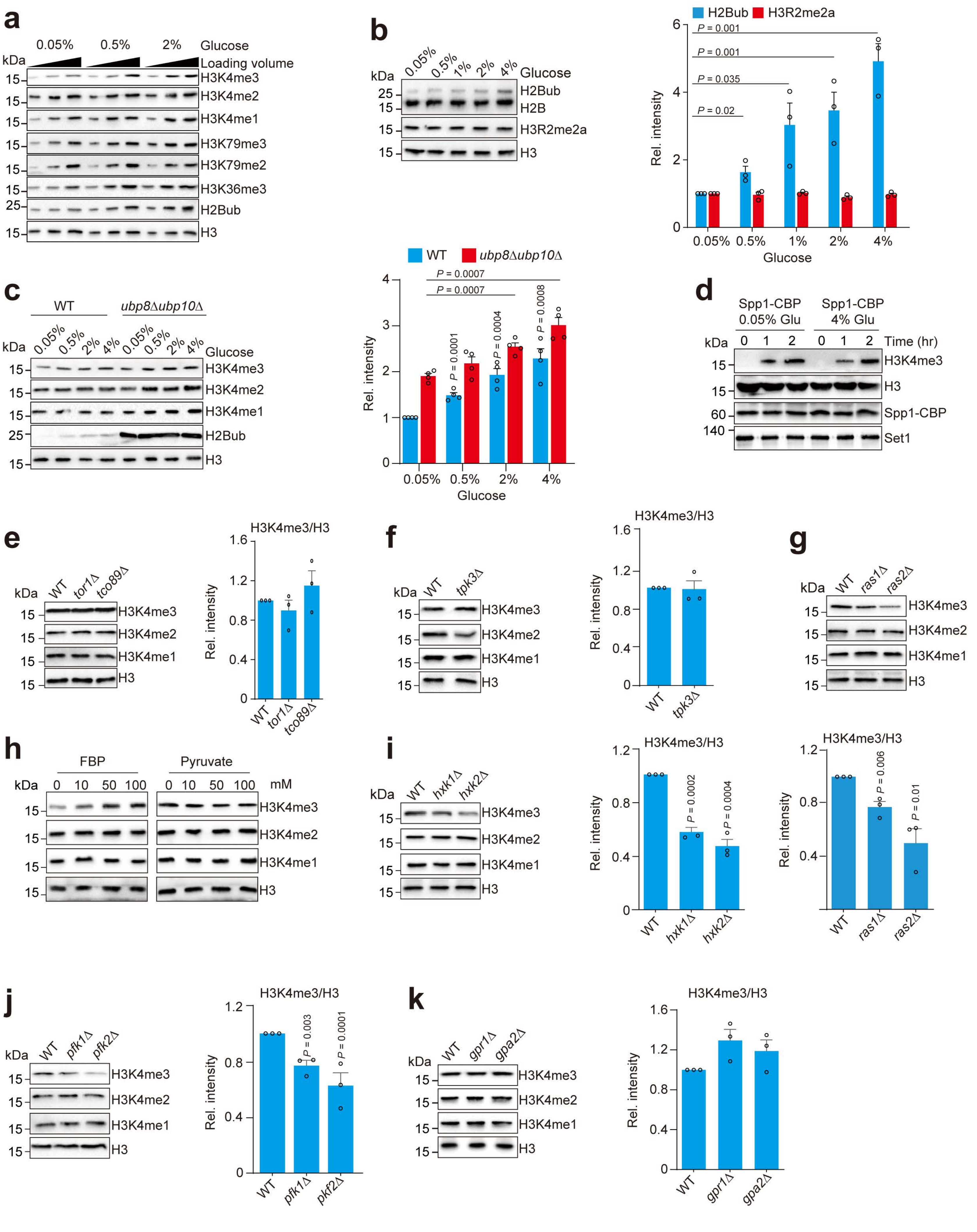

Original Supplementary Fig. 10

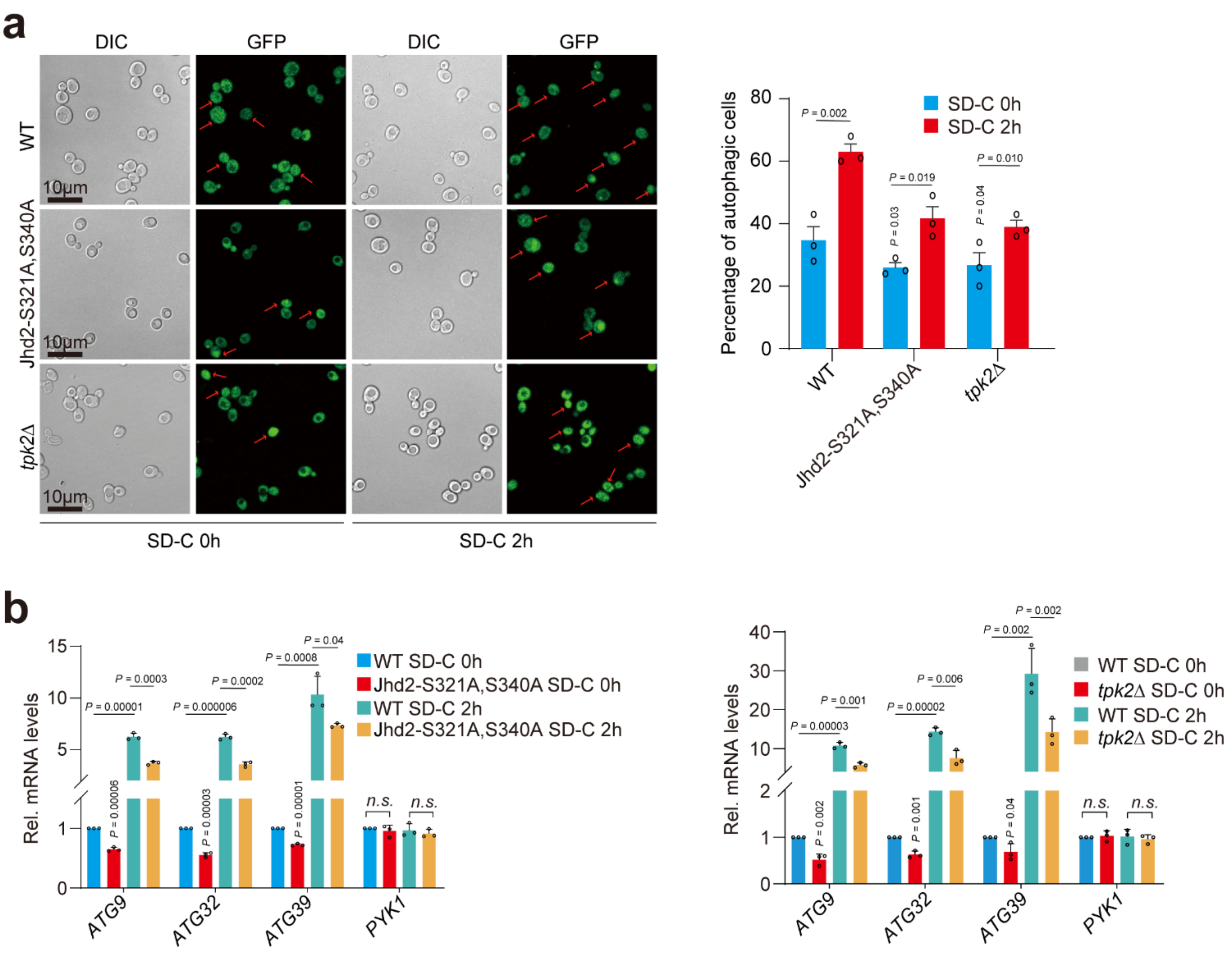

Supplementary Fig. 10 Tpk2-catalyzed Jhd2 phosphorylation promotes autophagy in part by inhibiting Rpd3 under glucose starvation conditions.

a Analysis of autophagy activity in WT, Jhd2-S321A, S340A, and *tpk2Δ* mutants when grown in glucose depletion medium (SD - C) for 0-2 hr by fluorescence assay. **b** Analysis of the transcription of *ATG9*, *ATG32*, and *ATG39* in WT, Jhd2-S321A, S340A, and *tpk2Δ* mutants when grown in glucose depletion medium (SD - C) for 0-2 hr by RT-qPCR.

For Supplementary Fig. 10a, b, data represent means ± SEM; n=3 independent experiments. Two-sided t-tests were used for statistical analysis.

Revised Supplementary Fig. 10

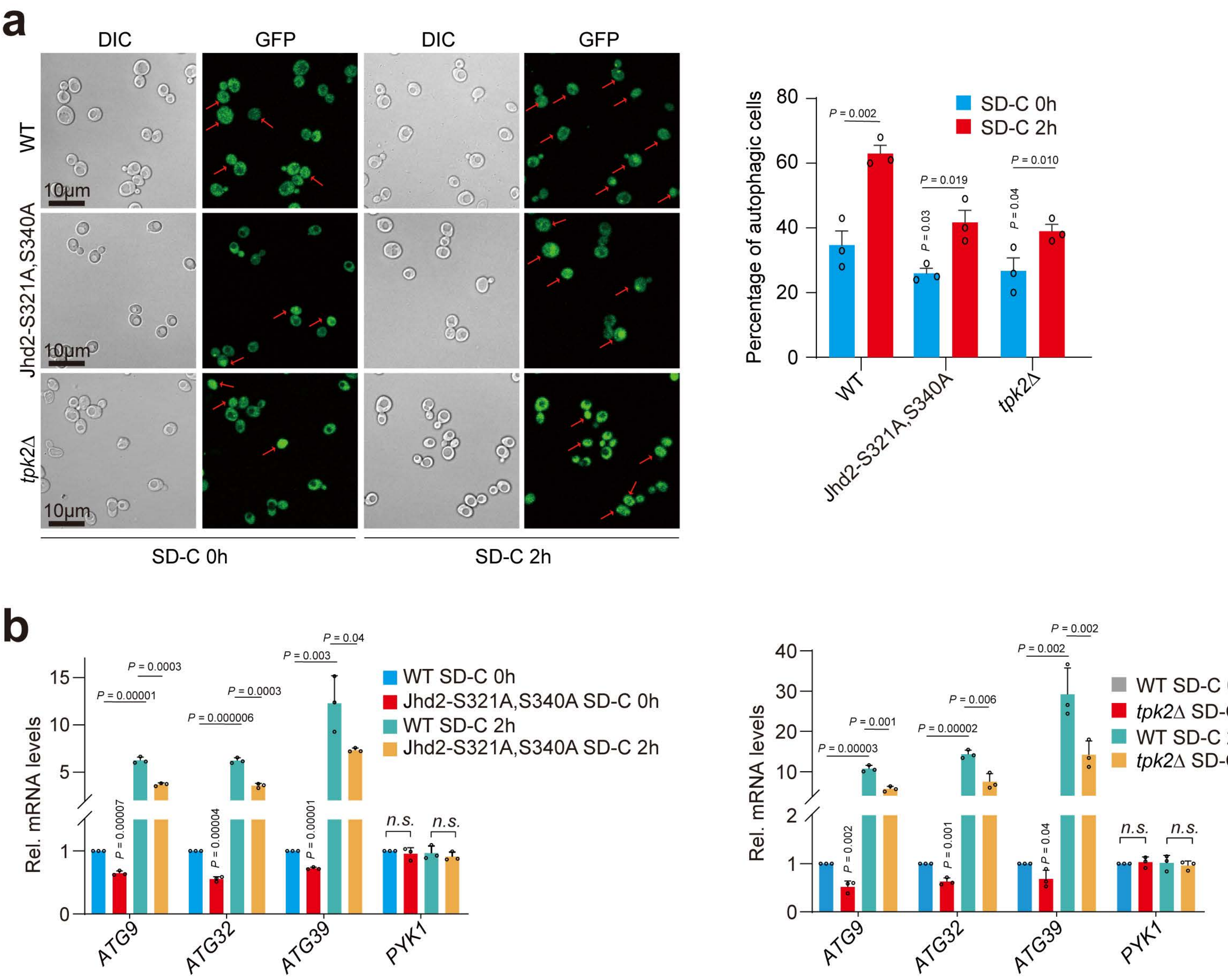

Supplementary Fig. 10 Tpk2-catalyzed Jhd2 phosphorylation promotes autophagy in part by inhibiting Rpd3 under glucose starvation conditions.

a Analysis of autophagy activity in WT, Jhd2-S321A, S340A, and *tpk2Δ* mutants when grown in glucose depletion medium (SD - C) for 0-2 hr by fluorescence assay. **b** Analysis of the transcription of *ATG9*, *ATG32*, and *ATG39* in WT, Jhd2-S321A, S340A, and *tpk2Δ* mutants when grown in glucose depletion medium (SD - C) for 0-2 hr by RT-qPCR.

For Supplementary Fig. 10a, b, data represent means ± SEM; n=3 independent experiments. Two-sided t-tests were used for statistical analysis.

Original Source Data
Supp Fig. 1g

	**WT**			***ras1Δ***			***ras2Δ***		
	**Rep1**	**Rep2**	**Rep3**	**Rep1**	**Rep2**	**Rep3**	**Rep1**	**Rep2**	**Rep3**
**H3K4me3**	1	1	1	0.688935	0.688935	0.833994	0.613742	0.613742	0.603002

	**Mean**			**SEM**			**P-value**	
	**WT**	***ras1Δ***	***ras2Δ***	**WT**	***ras1Δ***	***ras2Δ***	***ras1Δ/WT***	***ras2Δ/WT***
**H3K4me3**	1	0.737288	0.610162	0	0.048353	0.00358	0.005568	4.26E-08

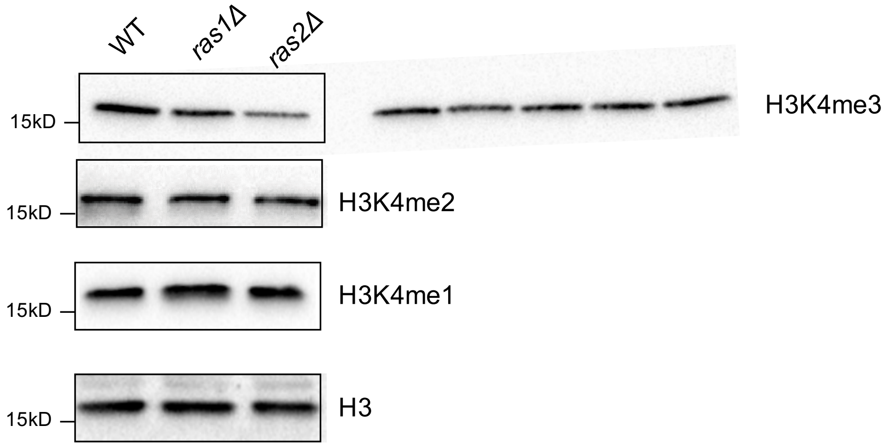

Revised Source Data
Supp Fig. 1g

	WT			*ras1Δ*			*ras2Δ*		
	Rep1	Rep2	Rep3	Rep1	Rep2	Rep3	Rep1	Rep2	Rep3
H3K4me3/H3	1	1	1	0.688935	0.790349	0.833994	0.613742	0.603002	0.281455

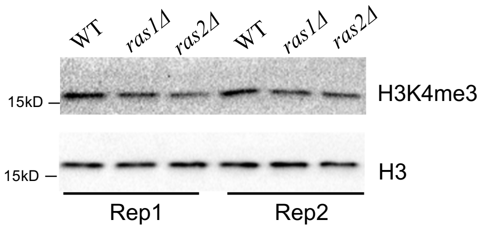

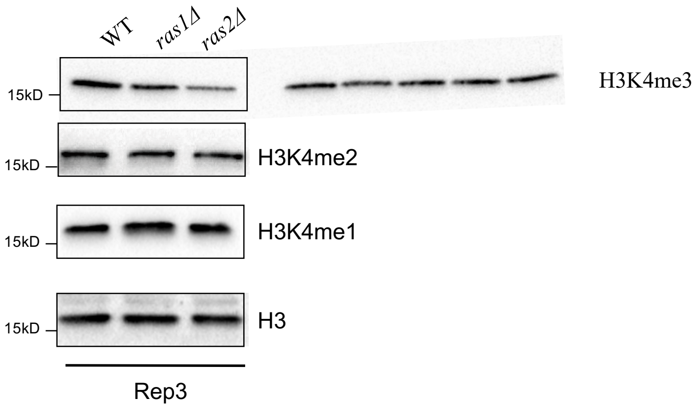

Supplement: Supplementary file 1 — Original and revised Fig. 7, Supplementary Figs. 1 and 10, Source Data Supplementary Fig. 1g [file 41467_2025_57545_MOESM1_ESM.pdf]
